# Focusing on the Cell Type Specific Regulatory Actions of NLRX1

**DOI:** 10.3390/ijms22031316

**Published:** 2021-01-28

**Authors:** Tünde Fekete, Dóra Bencze, Eduárd Bíró, Szilvia Benkő, Kitti Pázmándi

**Affiliations:** 1 Department of Immunology, Faculty of Medicine, University of Debrecen, 1 Egyetem Square, H-4032 Debrecen, Hungary; fekete.tunde@med.unideb.hu (T.F.); bencze.dora@med.unideb.hu (D.B.); 2 Doctoral School of Molecular Cell and Immune Biology, University of Debrecen, 1 Egyetem Square, H-4032 Debrecen, Hungary; biro.eduard@med.unideb.hu; 3 Department of Physiology, Faculty of Medicine, University of Debrecen, 98 Nagyerdei krt., H-4002 Debrecen, Hungary; benkosz@med.unideb.hu

**Keywords:** NLRX1, regulation, immune cells, myeloid cells, lymphoid cells, antiviral immunity, NF-κB pathway, autophagy, mitochondria, metabolism

## Abstract

Cells utilize a diverse repertoire of cell surface and intracellular receptors to detect exogenous or endogenous danger signals and even the changes of their microenvironment. However, some cytosolic NOD-like receptors (NLR), including NLRX1, serve more functions than just being general pattern recognition receptors. The dynamic translocation between the cytosol and the mitochondria allows NLRX1 to interact with many molecules and thereby to control multiple cellular functions. As a regulatory NLR, NLRX1 fine-tunes inflammatory signaling cascades, regulates mitochondria-associated functions, and controls metabolism, autophagy and cell death. Nevertheless, literature data are inconsistent and often contradictory regarding its effects on individual cellular functions. One plausible explanation might be that the regulatory effects of NLRX1 are highly cell type specific and the features of NLRX1 mediated regulation might be determined by the unique functional activity or metabolic profile of the given cell type. Here we review the cell type specific actions of NLRX1 with a special focus on cells of the immune system. NLRX1 has already emerged as a potential therapeutic target in numerous immune-related diseases, thus we aim to highlight which regulatory properties of NLRX1 are manifested in disease-associated dominant immune cells that presumably offer promising therapeutic solutions to treat these disorders.

## 1. Introduction

### 1.1. NLRs: A Large Receptor Family with Versatile Roles

Innate immunity serves as the first line of defense against invading pathogens. Cells of the innate immune system utilize a vast repertoire of evolutionary conserved pattern recognition receptors (PRRs) such as toll-like receptors (TLRs), C-type lectin receptors (CLRs), retinoic acid-inducible gene (RIG)-I-like receptors (RLRs) and nucleotide-binding oligomerization domain (NOD)-like receptors (NLRs) to detect various microbial motifs or danger signals [1]. Therefore, they play a central role in the initiation of host defense during the early stages of infection and subsequently contribute to the generation of adaptive immune responses as well [2].

Among PRRs, NLRs comprise a large family of intracellular receptors/sensors, which play an essential role in recognition of a wide variety of pathogen-associated molecular patterns (PAMP) and exogenous/endogenous damage-associated molecular patterns (DAMP), and eventually lead to the induction of innate immune effector functions. Besides, NLRs have been recognized to be involved in various cellular processes as positive or negative regulators. To date, 22 NLRs have been identified in humans, and at least 34 in mice [3]. Based on their unique function, they are classified into five subgroups:

(1) The most thoroughly characterized NLRs form large multiprotein complexes, termed inflammasomes, the activation of which leads to the cleavage of pro-IL-1ß and pro-IL-18 pro-inflammatory cytokines into their mature, biologically active form. Among NLRs, NLRP1, NLRP3, NLRP6 and NLRC4 are known to form inflammasomes.
(2) Several NLRs act as positive or negative regulators of signal transduction. The so-called regulatory NLRs control various intracellular signaling cascades such as the nuclear factor-kappa B (NF-κB), the type I interferon (IFN) and the mitogen-activated protein kinase (MAPK) pathways initiated by other PRRs. This group is composed of several members such as NOD1, NOD2, NLRC5, nucleotide-binding domain and leucine-rich repeat–containing protein X1 (NLRX1), NLRP12 and NLRC3.
(3) A specific subgroup of NLRs, including NLRC5 and the class II major histocompatibility complex transactivator (CIITA), can function as enhanceosomes and control the transcription of MHC I and II genes, respectively.
(4) Upon bacterial sensing, some NLRs, such as NOD1 and NOD2, are able to recruit the ATGL16L1 autophagy modulating protein to the cell membrane to initiate the formation of autophagosomes [4].
(5) Reproductive NLRs (NLRP2, NLRP5, NLRP7) control embryogenesis and reproduction. The function of these NLRs has been extensively reviewed in reference [5].

Regarding the structure of NLRs, they all share a common C-terminal leucine-rich repeat (LRR) domain, which are responsible for ligand recognition; a central NOD, also called as NACHT domain, which upon nucleotide binding mediates the activation and oligomerization of the receptor; and an N-terminal domain, which binds to distinct adaptor proteins and exerts multiple downstream signaling. Based on their N-terminal effector domains, NLRs are divided into four subfamilies:

(1) The NLRA subfamily possesses an acidic transactivation domain (TA) and consists of only one member, the CIITA.
(2) The NLRB subfamily has a baculoviral inhibition of apoptosis protein repeat (BIR) domain. Similar to NLRA subfamily, NLRB subfamily has also only one member, the NLR family apoptosis inhibitory protein (NAIP).
(3) The NLRC subfamily owns a caspase recruitment and activation domain (CARD), and in contrast to the above mentioned two families, the NLRC subfamily consists of six receptors: NOD1 (or NLRC1), NOD2 (or NLRC2), NLRC3, NLRC4, NLRC5, and NLRX1.
(4) The NLRP subfamily has a pyrin domain (PYD), and comprising 14 members represents the largest subfamily of NLRs: NLRP1, NLRP2, NLRP3, NLRP4, NLRP7, NLRP12, NLRP5, NLRP6, NLRP8, NLRP9, NLRP10, NLRP11, NLRP13, and finally NLRP14 (reviewed in reference [4]).

### 1.2. Beyond the Inflammasomes: Regulatory NLRs

The inflammasome forming NLRs represent without doubt the most studied subgroup of the NLR family. However, due to their diverse functions, regulatory NLRs have also landed at the forefront of research in the field of immunology. The functions of regulatory NLRs have already studied in various immune cells such as macrophages or human dendritic cells (DCs), since these receptors can modulate diverse signaling pathways initiated by other PRRs and can shape immune cell mediated responses. Inflammatory reactions must be tightly controlled to keep the immune response in balance. On one hand, the host must mount an immune response strong enough to clear the pathogen from the body, on the other hand, the immune response must be fine-tuned to prevent excessive collateral damage. Modulation of the inflammatory cascades such as NF-κB, MAPK and type I IFN pathways has been already documented by these receptors [6]. Regulatory NLRs may act synergistically or antagonistically with other PRRs, that seems to depend on the type of cell, receptor or ligand involved.

For instance, the bacterial sensors, NOD1 and NOD2 are positive regulatory NLRs, as they enhance inflammatory responses. Upon ligand sensing, NOD2 oligomerizes and recruits distinct proteins resulting in the formation of a multiprotein complex, termed the NODosome. Receptor-interacting-serine/threonine-protein kinase 2 (RIP2), mitochondrial antiviral signaling protein (MAVS), NF-kappa-B essential modulator (NEMO) and X-linked inhibitor of apoptosis (XIAP) are some of the few recruited proteins, which can affect downstream signaling events. It has been described that the NODosome promotes type I IFN signaling via either TNF receptor-associated factor (TRAF) 3/ IFN regulatory factor (IRF) 7 or MAVS/IRF3 and modulates NF-κB signaling as well [6].

In contrast, NLRC3 and NLRP12 are negative regulatory NLRs, the major function of which is to prevent unwanted inflammation. Similar to the inflammasome and the NODosome, these NLRs are also capable of forming multiprotein complexes, termed TRAFosomes. NLRC3 interacts with TRAF6, while NLRP12 binds to TRAF3. NLRC3 inhibits NF-κB signaling by decreasing the overall ubiquitination of TRAF6. In addition, NLRC3 negatively regulates type I IFN responses by interfering with the stimulator of interferon genes (STING)/TANK-binding kinase 1 (TBK1) axis via directly binding to STING and TBK1 [7]. NLRP12 is the negative regulator of canonical and non-canonical NF-κB signaling and plays a role in inhibiting the extracellular signal-regulated kinase (ERK) pathway as well [8].

Besides the abovementioned receptors, NLRC5 has emerged as a regulatory NLR, which affects the TLR- and RLR-mediated NF-κB and type I IFN signaling negatively [9,10,11]. Fekete et al. also described that NLRC5 suppresses the RLR-induced type I IFN response in plasmacytoid DCs (pDC) similar to NLRX1, which is another important regulatory NLR [12]. NLRX1 has been primarily described as a negative regulator of inflammatory reactions, but soon conflicting data have been reported pertaining to its role in immune responses. The observed discrepancies might be attributed to the different cell types, and the opposing regulatory effects of NLRX1 are most striking when immune cells are compared to non-immune cells. Therefore, in this review we would like to focus on the regulatory function of NLRX1 in a cell type specific manner.

### 1.3. X Marks the Spot: NLRX1, a Mitochondria-Associated Regulatory NLR

**Figure 1:**
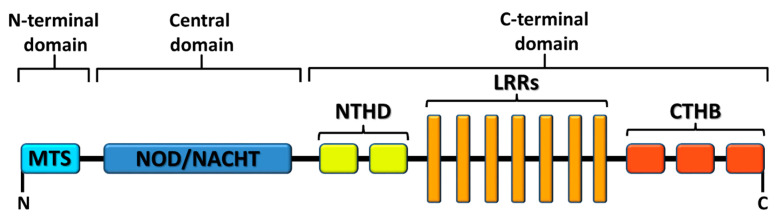
Schematic representation of NLRX1 structure. CTHB: C-terminal three-helix bundle; LRRs: leucine-rich repeats; MTS: mitochondrial targeting sequence; NACHT: domain conserved in NAIP, CIITA, HET-E and TP1; NOD: nucleotide-binding oligomerization domain; NTHD: N-terminal helical domain.

NLRX1, also known as NOD5, NOD9, or CLR11.3 belongs to the subfamily of NLRC receptors. It consists of 975 amino acids and is expressed ubiquitously. “X” in algebra is often used to signify the unknown. Similarly, the X in NLRX1 refers to its enigmatic structure, more precisely to its not fully characterized N-terminal domain. Instead of the commonly shared CARD domain of NLRC receptors, only one domain was identified in the N-terminus, the mitochondrial targeting sequence (MTS), which uniquely localizes NLRX1 to the mitochondrial membrane [13]. It was initially reported, that NLRX1 is associated with the mitochondrial outer membrane [14]. Later, it was shown that NLRX1 can also localize to the inner mitochondrial membrane and within the matrix, and last but not least in the cytosol. All these data suggest, that similar to other NLRs, NLRX1 can shuttle between distinct cellular compartments, from the cytosol to the mitochondria that makes it possible to interact with a multitude of cellular pathways [6]. The C-terminal domain of NLRX1 displays a three-domain architecture [15]. It consists of an N-terminal helical domain, central seven LRRs and a C-terminal three-helix bundle, essential for structural integrity. It has been revealed that the C-terminal domain of NLRX1 can form hexamers by the trimerization of dimers both in solution and in crystal structures [16]. Hong et al. also found that NLRX1 can directly interact with RNA ligands but not DNA probably through a positively charged surface area of the C-terminal fragment. Similar to other NLRs, it contains a central NOD/NACHT domain as well [15] (Figure 1).

NLRX1 has diverse cellular functions depending on its localization and interacting partners. NLRX1 also serves as a scaffold protein and is able to take part in the assembly of large multiprotein complexes [17]. In addition, it has been documented that NLRX1 regulates the NF-κB and type I IFN signaling, affects the MAPK pathway, modulates ROS production, influences the main metabolic pathways and impacts autophagy and cell death. Besides regulating host-pathogen interactions, NLRX1 acts as a tumor suppressor or, on the contrary, might facilitate metastasis development. More importantly, it has a protective role in the development and progression of various autoimmune diseases such as multiple sclerosis, lupus and inflammatory bowel disease. Furthermore, NLRX1 has also been found to contribute to metabolic disorders. The importance of NLRX1 in inflammatory, metabolic and oncological diseases have been already addressed in previous and recent reviews [17,18]. Since NLRX1 contributes to a multitude of diseases, targeting NLRX1 could offer promising treatment strategies in immune cell-, or non-immune cell-mediated diseases [18].

Before discussing the cell type specific differences in NLRX1 function, in the next section we briefly review the underlying molecular mechanisms behind the complex regulatory role of NLRX1, which have been identified to date.

## 2. Regulatory Mechanism behind the Multiple Actions of NLRX1

### 2.1. Regulation of Antiviral Immunity

To detect replicating viruses, cells utilize cytosolic RLRs including RIG-I and melanoma differentiation-associated protein 5 (MDA5), which are specialized for sensing viral replication intermediates in the cytoplasm. RLRs use the mitochondrial adaptor MAVS to stimulate type I IFN responses. MAVS contains an N-terminal CARD domain, through which it interacts with RIG-I. Moore et al. elegantly proved in an in vitro system using HeLa and HEK293T cells that NLRX1 is a negative regulator of RIG-I, and possibly MDA-5-induced antiviral signaling by competing with the receptors for the CARD domain of MAVS. They reported that NLRX1 is located on the mitochondrial outer membrane and similar to RIG-I, it can bind the CARD domain of MAVS. Via this interaction NLRX1 negatively regulates Sendai virus-induced type I IFN production by inhibiting MAVS mediated IRF3 dimer formation [14]. Under in vivo conditions, the negative regulatory role of NLRX1 in virus induced inflammation was proved by Allen et al. in a mouse model of influenza infection. However, NLRX1^−/−^ mice cleared the virus more quickly, exhibited enhanced levels of IL-6 and IFN-β, and showed more severe lung injury and morbidity [19].

Apart from direct competition for MAVS binding, NLRX1 can indirectly inhibit RIG-I-MAVS signaling as well. In a human hepatoma cell line, it was demonstrated that the nucleotide-binding domain of NLRX1 attenuates hepatitis C virus (HCV)-triggered RIG-I-MAVS signaling by recruiting poly(rC) binding protein 2 (PCBP2) to MAVS. PCBP2 induces K48-linked polyubiquitination and subsequent proteasomal degradation of MAVS, thus it limits type I IFN production [20]. Furthermore, NLRX1 can also be used as pro-viral host factor by many other viruses to modulate the host’s antiviral response and aid virus replication and survival. For example, it was published that in Rhesus monkeys simian immunodeficiency virus (SIV) triggers NLRX1 expression in the beginning of the infection to facilitate its own replication [21]. It was also demonstrated that ORF9c protein of SARS-CoV-2 interacts with NLRX1, through which it might influence MAVS-mediated type I IFN and pro-inflammatory cytokine secretion [22].

NLRX1-mediated inhibition of the antiviral immune response can be overcome with the help of FAS-associated factor-1 (FAF1), a member of the FAS death-inducing signaling complex. Besides being involved in FAS-mediated apoptosis, FAF1 can directly bind to NRLX1 thereby freeing MAVS and enabling RIG-I-MAVS interaction. Thus FAF1 positively regulates type I IFN production of immune cells in response to RNA viruses, and its inhibitory effect on NLRX1 further proves the negative regulatory role of NLRX1 in MAVS-mediated responses [23].

Besides regulating the RIG-I-MAVS-induced type I IFN signaling, NLRX1 controls the STING and TBK1-mediated pathways as well. NLRX1, by binding the DNA sensor STING, disrupts STING-TBK1 signaling and thereby suppresses TBK1 activation, which is required for type I IFN production in response to stimuli like human immunodeficiency virus (HIV)-1, DNA viruses, cGAMP and dsDNA in myeloid cells. In line with this, NLRX1 deficient mice are more resistant to HIV-1 and DNA viruses [24]. Interestingly, in contrast to IRF3 pathway, NLRX1 positively regulates the NF-κB-driven IRF1-mediated early antiviral responses in hepatocytes suggesting the opposing regulatory role of NLRX1 in the control of IRF3 and IRF1 pathways. In hepatocytes, NLRX1 competes with protein kinase R (PKR) for viral RNA binding, which prevents the PKR induced blockade of host’s protein synthesis. Thus, NLRX1 helps to maintain IRF1 upregulation, while inhibits IRF3 dimerization upon viral infection [25].

Other studies also showed that NLRX1 can positively control the innate antiviral immune responses via interaction with viral proteins. Influenza A virus expresses a small protein, polymerase basic protein 1-frame 2 (PB1-F2), which induces apoptosis in innate immune cells by disrupting mitochondrial membrane potential. In macrophages, NLRX1 promotes type I IFN production and protects macrophages from influenza-induced apoptosis by binding to the viral PB1-F2. Similarly, NLRX1^−/−^ mice infected with influenza displayed impaired type I IFN response, increased viral replication in the lung, and increased airway hyperreactivity [26].

**Figure 2:**
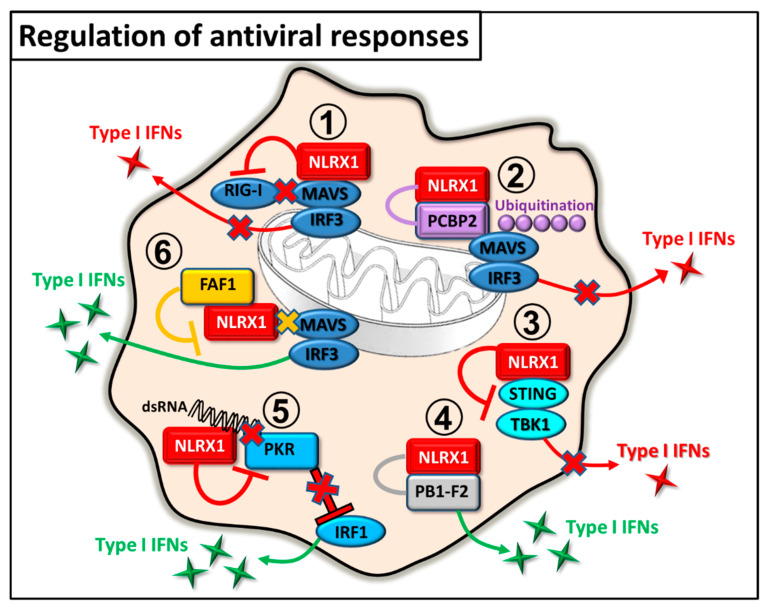
Regulation of antiviral responses by NLRX1. 1. NLRX1 inhibits RIG-I binding to MAVS, resulting in decreased type I IFN response. 2. NLRX1 recruits PCBP2 to MAVS and thereby triggers its ubiquitination and degradation that leads to reduced type I IFN levels. 3. NLRX1 prevents TBK1-mediated type I IFN secretion by binding to STING. 4. NLRX1 interacts with PB1-F2 and promotes type I IFN production. 5. NLRX1 competes with PKR for binding to double stranded RNA and prevents the PKR-driven blockade of host’s protein synthesis, which promotes the upregulation of IRF1-mediated early antiviral responses. 6. FAF1 interacts with NLRX1 and blocks the binding of NLRX1 to MAVS that positively effects the MAVS-mediated type IFN responses.

The previous results clearly indicate that the mode of NLRX1-mediated regulation in antiviral responses is cell type specific and mainly determined by the features of the invading viruses, which can directly or indirectly modulate the function of NLRX1 (Figure 2). By negatively regulating antiviral immune responses, NLRX1 not only makes the host susceptible to viral infections but promotes virus-induced tumor development (e.g., Kaposi’s sarcoma or primary effusion lymphoma) as well [27]. On the other hand, due to the potential detrimental and autoimmune promoting effects of sustained type I IFN levels, antiviral immune responses must be tightly controlled. Thus, it seems that NLRX1, at least partially, is responsible to keep this delicate balance under control.

### 2.2. Regulation of NF-κB Pathway

In 2011, studies from two different research groups were published in the same issue of Immunity stating that besides regulating RIG-I and STING signaling pathways, NLRX1 is also capable to modulate TLR signaling in vivo, and influence NF-κB-driven pro-inflammatory responses. Allen et al. studied the role of NLRX1 in the TLR signaling of mice and found that, upon lipopolysaccharide (LPS) stimulation, NLRX1^−/−^ macrophages produced higher amounts of IFN-β and IL-6 compared to wild type macrophages. Intratracheal instillation with LPS significantly increased IL-6 levels and inflammation in the lung of NLRX1^−/−^ mice compared to wild type animals. Furthermore, their results suggest that NLRX1 suppresses NF-kB activation by interacting and interfering with TRAF6, which regulates a diverse range of innate immune signaling pathways, including TLR4 [19].

In line with these findings, Xia et al. demonstrated that NLRX1 interacts with TRAF6 in various unstimulated cells types including mouse embryonic fibroblasts (MEF), THP-1, RAW264.7 and 293T cells. However, upon TLR4 stimulation, both NLRX1 and TRAF6 rapidly undergo K63-linked polyubiquitination that results in dissociation of NLRX1 from TRAF6. Once NLRX1 is free, it targets the IKK complex via its LRR domain and impairs IKKα/β phosphorylation and kinase activity for IκBα phosphorylation, resulting in attenuated NF-κB activation and pro-inflammatory cytokine production. Furthermore, higher levels of IL-6 were detected in NLRX1 deficient mice, which were more susceptible to LPS induced septic shock compared to wild type animals [28].

Another possible mechanism for hindering pro-inflammatory responses by NLRX1 is the sequestration of pro-inflammatory molecules. In an in vitro model of mitochondrial injury pro-inflammatory mediators, including MAVS, p-TBK1, p-IKK, IκB, and TRAF6 were recruited to the mitochondria, where they interacted with NLRX1. The lack of NLRX1 resulted in increased NF-κB and TBK1 mediated immune response and apoptosis in rat pulmonary microvascular endothelial cells. These data suggest that NLRX1 may have a role in the maintenance of cellular homeostasis after acute injury by sequestering pro-inflammatory molecules [29]. In addition to negatively regulating NF-κB signaling, NLRX1 also serves as a tumor suppressor [30,31].

**Figure 3:**
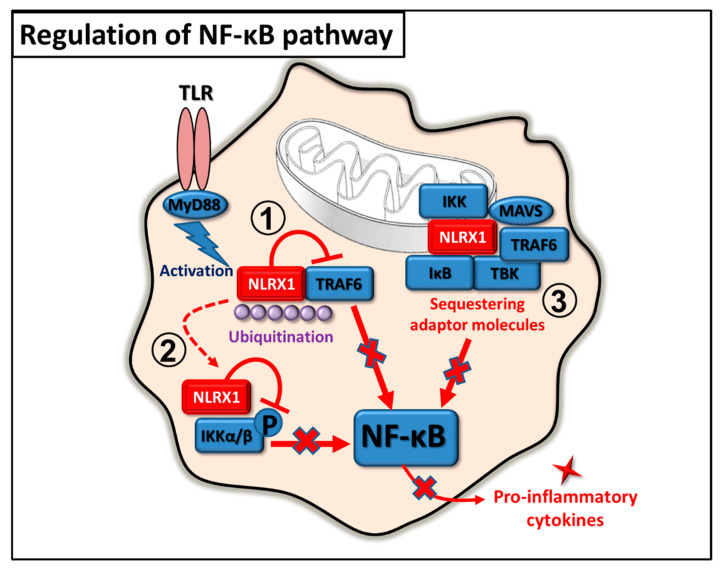
Regulation of NF-κB pathway by NLRX1. 1. In inactivated cells, NLRX1 interacts with TRAF6 and inhibits the NF-κB pathway. 2. In activated cells, NLRX1 dissociates from TRAF6 via polyubiquitination and binds to IKK complex to prevent NF-κB-mediated pro-inflammatory cytokine production. 3. Upon acute injury, activated MAVS, TBK1, IKK, IκB, and TRAF6 are recruited to the mitochondrion, where they interact with NLRX1, which blocks NF-κB activity.

The above findings demonstrate that during pro-inflammatory immune responses the main function of NLRX1 is to prevent overzealous inflammatory responses, which can promote unwanted tissue damage in the host (Figure 3).

### 2.3. Regulation of Autophagy

Autophagy strongly associates with antiviral immunity since it can shape antiviral immune responses by exerting either antiviral or proviral effects depending on the type of the host cell and the invading virus. In 2012, Lei et al. identified a mitochondrial NLRX1 interacting protein, the Tu translation elongation factor (TUFM), which can also engage RIG-I and the autophagy-related protein (ATG) 5-ATG12 conjugate and ATG16L1. It was described that NLRX1 and TUFM inhibits RIG-I-induced type IFN signaling in MEFs. Moreover, NLRX1 and TUFM were found to be essential for vesicular stomatitis virus (VSV)-induced autophagy in MEFs and mouse peritoneal macrophages. These findings indicate that NLRX1 and TUFM oppositely regulate type I IFN production and autophagy: their interaction is required to ensure optimal levels of autophagy and, at the same time, to keep IFN production in balance during viral infection [32]. The same research group described that the mitochondrial NLRX1-TUFM complex not only has a role during viral infection but also promotes autophagy and survival of tumor cells [33]. Furthermore, the oncogenic human papillomavirus 16 (HPV16) also exploits the autophagy inducing capacity of NLRX1 via its E7 oncoprotein, which can interact with the mitochondria-located NLRX1 [34].

In contrast to viral infection, it was also observed that NLRX1 negatively regulates autophagy upon bacterial infections caused by *Group A Streptococcus (GAS)*. After GAS invasion, the bacteria escape from the endosome to the cytosol, where they are rapidly degraded by autophagy. However, it was shown that NLRX1 interacts with the Beclin 1-UV radiation resistance-associated gene protein (UVRAG) complex via its NACHT domain to negatively regulate GAS-induced autophagy in HeLa cells [35].

Apart from canonical autophagy, NLRX1 also promotes the non-canonical form of autophagy, termed microtubule-associated protein 1 light chain 3 (LC3)-associated phagocytosis (LAP) upon fungal infection. In *Histoplasma capsulatum*-activated macrophages, NLRX1 forms a complex with TUFM-ATG5-ATG12 and facilitates the incorporation of LC3-II into the phagosomal membrane that ensures its maturation. In addition, the NLRX1-TUFM interaction can also enhances the pro-inflammatory cytokine production of macrophages by the activation of MAPK signaling upon fungal stimulation [36].

**Figure 4:**
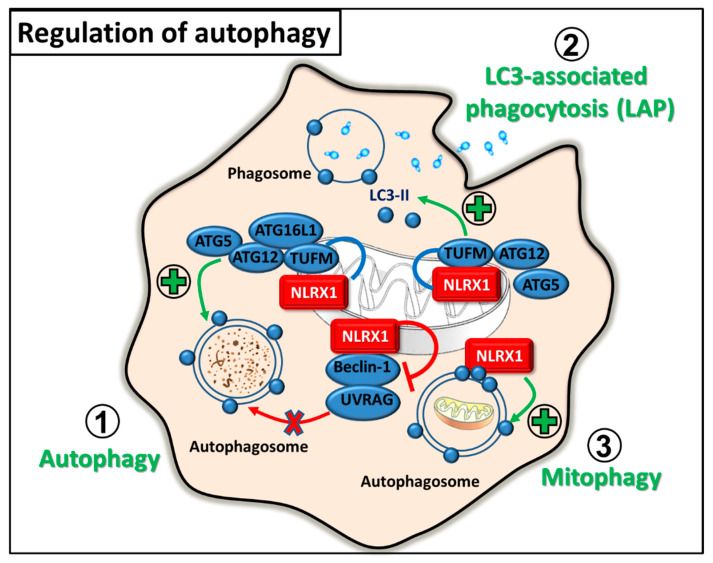
Regulation of autophagy by NLRX1. 1. In virus-infected or tumour cells, NLRX1 interacts with TUFM and promotes autophagy or, conversely, inhibits Beclin-1-UVRAG complex to reduce autophagosome formation upon bacterial infection. 2. Upon fungal infection, NLRX1-TUFM complex formation aids LC3-associated phagocytosis by facilitating the incorporation of LC3-II to the phagosomal membrane. 3. Via interaction with LC3, NLRX1 recruits autophagosomes to the mitochondria and promotes mitophagy.

Furthermore, NLRX1 is essential for mitophagy, a selective form of autophagy, which ensures the clearance of damaged or dysfunctional mitochondria. NLRX1 contains an LC3-interacting region (LIR), through which it is able to recruit autophagosomes to the mitochondria, thus NLRX1 was described as a novel mitophagy receptor. Intracellular pathogens, for example *Listeria monocytogenes* can activate mitophagy via NLRX1 and promote their survival by reducing mitochondrial reactive oxygen species (mtROS) production. In bone marrow-derived macrophages (BMDM), *Listeria* induces mitophagy via its listeriolysin O (LLO) component, which frees the NACHT domain of NLRX1 from the LRR-maintained autoinhibited state and allows oligomerization and subsequent binding to LC3-decorated autophagosomes [37]. Besides pathogen-induced mitophagy, NLRX1 also regulates mitophagy in mammary tumors, since NLRX1 upregulation in aggressive metastatic breast cancer cell lines is associated with higher metastatic potential. Higher level of NLRX1 expression is required for maintaining increased levels of TNF-α-induced autophagy, which ensures mitochondrial homeostasis by eliminating dysfunctional mitochondria. In line with this, NLRX1 deficiency impairs mitochondrial metabolic function and the lysosomal turnover of mitochondria, which in turn decreases the migration and proliferation of aggressive triple-negative breast cancer cells [38]. Overall, these data implicate that targeting NLRX1 could offer promising strategies in the therapy of autophagy-related diseases (Figure 4).

### 2.4. Regulation of ROS Production and Cell Death

The regulatory actions of NLRX1 are mainly associated with mitochondria. In HEK293T cells, it was observed that NLRX1 can be also localized within the mitochondrion, at the matrix side of the mitochondrial inner membrane, where it can interact with UQCRC2, a member of complex III of mitochondrial electron transport chain (ETC). Through interaction with UQCRC2, which is essential for mtROS production, NLRX1 is also capable to control mtROS-dependent cellular functions including ROS sensitive pro-inflammatory signaling pathways or cell death [13]. ROS have either protective or detrimental effects upon infections, and the ability of NLRX1 to modulate mtROS production can also be hijacked by many invading pathogens [39,40].

In airway epithelial cells, it was shown that NLRX1 is expressed in both the cytoplasm and at the apical surface; however, after infection it translocates to the mitochondria. Upon sensing rhinovirus RNA or synthetic polyinosinic:polycytidylic acid [poly (I:C)], the mitochondria-associated NLRX1 promotes mtROS production, which is required for virus-induced epithelial barrier disruption [39]. Some intracellular bacteria take advantage of the ROS producing capacity of the host cell as well. For instance, *Chlamydia trachomatis* utilizes ROS to activate caspase-1 in epithelial cells [40]. Whereas in macrophages caspase-1 initiates the secretion of high amounts of IL-1β, in epithelial cells, which produce only negligible amounts of IL-1β, it rather enhances lipid metabolism [41]. *Chlamydia* requires host-derived lipids for intracellular growth since it replicates within a specialized membrane-bound compartment termed the inclusion [42]. Thus, the increased lipid metabolism is a requirement for optimal growth and stability of the inclusion membrane as well as for chlamydial replication. It was found that, at first, *Chlamydia* initiates ROS production by activating NADPH oxidases, then it also exploits the mtROS inducing capacity of NLRX1 in HeLa cells. Consequently, the increased levels of intracellular ROS stimulate the NLRP3 dependent caspase-1 activation, which ensures the survival of the pathogen [40].

Upon infections, cells often use the inflammation promoting features of ROS that can facilitate immune responses against pathogens. In HeLa cells, overexpression of NLRX1 increased the TNF-α, *Shigella flexneri* and poly (I:C)-induced ROS production, which potentiated the NF-κB and c-Jun N-terminal kinase (JNK) pathways. The authors speculate that the positive regulatory effect of NLRX1 on ROS production was independent of its N-terminal domain, but rather the NACHT or LRR domains of the receptor might be involved in ROS generation [43].

The regulatory role of ROS can be also observed in the context of cell death. In a model of cisplatin-induced ototoxicity, it was reported that NLRX1 promotes cisplatin-induced mitochondrial apoptosis by potentiating ROS/JNK signaling upon stimulating the auditory cells with cisplatin [44]. In addition, cisplatin insult upregulated NLRX1 expression and autophagy, which consequently triggered cell death in auditory cells [45]. Furthermore, it was also reported that NLRX1 can sensitize HEK293 cells to TNF-α-induced apoptosis through interaction with the mitochondria-localized caspase-8 and induction of mtROS production. This observation reflects the tumor suppressor feature of NLRX1 [46].

In contrast, depending on the cell type, NLRX1 can also play a protective role and can prevent cell death. It has been demonstrated that NLRX1 functions as a negative regulator of LPS-induced NF-κB signaling, and hinders inflammation and apoptosis in chondrocytes [47]. In addition, another study revealed that NLRX1 protects tubular epithelial cells from mitochondrial injury and apoptosis by decreasing oxidative stress in a mouse model of renal ischemia-reperfusion injury [48].

**Figure 5:**
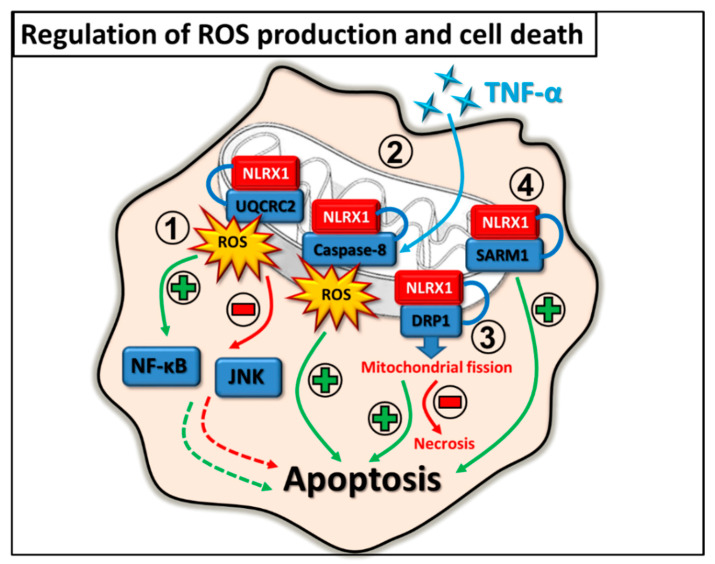
Regulation of ROS production and cell death by NLRX1. 1. NLRX1 interacts with UQCRC2 and can either induce or inhibit mtROS production in a cell type dependent manner. Through regulating mtROS production, NLRX1 might have either a positive or negative effect on NF-κB and JNK inflammatory pathways and on the induction of apoptosis. 2. NLRX1 positively regulates the TNF-α-induced caspase-8 dependent apoptosis via the interaction with the mitochondrial localized caspase-8. 3. NLRX1 binds to DRP1, and thus promotes mitochondrial fission, inhibits necrosis while supporting apoptosis. 4. NLRX1 interacts with SARM1 and promotes SARM1-dependent apoptosis.

Interestingly, regulation of cell death by NLRX1 is not always dependent on ROS production. It was reported that upon rotenone treatment, overexpression of NLRX1 increased the viability of mouse neuroblastoma cells in an ROS independent manner. The authors observed that NLRX1 interacts with GTPase dynamin-related protein 1 (DRP1), which has an essential role in regulating mitochondrial dynamics to promote mitochondrial fission and thus rescues cells from necrosis. In this situation, NLRX1 seems to support apoptosis rather than necrosis [49]. In addition, in non-neuronal cells, NLRX1 has another interacting partner, the sterile alpha and toll/interleukin-1 receptor (TIR) motif- containing protein 1 (SARM1), which plays a fundamental role in cell homeostasis and regulation of cell death. Via the interaction with SARM1, NLRX1 can influence the SARM1 dependent apoptosis in HEK293T [50] (Figure 5).

### 2.5. Regulation of Metabolism

Metabolic reprogramming of cells, and targeting the main metabolic pathways can be a promising therapeutic tool for a wide variety of diseases. NLRX1 is considered to be an emerging metabolic regulator that can control cancer cell metabolism and has the potential to alleviate the symptoms of autoimmune diseases as well.

In the literature, there are conflicting data regarding the regulatory role of NLRX1 in cell metabolism. The observed discrepancies point to the highly cell type specific nature of NLRX1 actions. Due to its mitochondrial localization and its interaction with the ETC components such as UQCRC2, a member of complex III [13], NLRX1 can be a potential regulator of oxidative phosphorylation (OXPHOS). So far, it seems that NLRX1 can promote OXPHOS in immune cells [51,52], while supports aerobic glycolysis and attenuates OXPHOS in cancer or non-immune cells [46,53,54,55]. Cancer cells are characterized by high glycolytic activity and it was observed that the inhibition of glycolysis is associated with decreased NLRX1 expression [53]. Furthermore, NLRX1 impaired the activity of mitochondrial ETC components and supported aerobic glycolysis in tumour cells [46]. In addition, Sing et al. described that NLRX1 is also present in mitochondrial RNA granules, where the post-transcriptional processing of de novo synthetized mitochondrial RNA and ribosome biogenesis occur under the control of an RNA-binding protein, the Fas-activated serine-threonine kinase family protein-5 (FASTKD5). NLRX1 can bind to FASTKD5 and mitochondrial RNA via its LRR domain, and inhibit the maturation of precursor transcripts for complexes I and IV. In the presence of NLRX1, the assembly and activity of respiratory chain complexes are attenuated resulting in decreased OXPHOS activity [54]. Moreover, NLRX1 attenuates fatty acid-dependent OXPHOS and enhances glycolysis in hepatocytes [55].

Contrary to the above findings, NLRX1 rather promotes OXPHOS in immune cell-related diseases. In inflammatory bowel disease (IBD) models, NLRX1 deficiency led to a metabolic switch towards aerobic glycolysis, which resulted in enhanced inflammation. NLRX1 deficient CD4+ T cells differentiated more likely into helper T (Th) 17 cells than wild type cells, and displayed enhanced proliferation rates. This was the consequence of enhanced aerobic glycolysis caused by increased lactate dehydrogenase (LDH) activity and incomplete fatty acid oxidation due to the lack of NLRX1. Interestingly, the expression of the hypoxia-inducible factor 1-alpha (HIF-1α) transcription factor, which controls LDH activity and Th17 differentiation is also increased in the absence of NLRX1 [51]. One of the promising NLRX1 agonists in IBD is NX-13, a gut-restricted selective NLRX1 activator, which targets metabolic regulation through NLRX1. In IBD models, attenuated LDH activity, glucose uptake, NF-kB activity and ROS production were observed in the presence of NX-13, and cells were rather characterized by enhanced OXPHOS [52].

NLRX1 also has a potential to regulate the glutamine metabolism of the gut. It is a well-known fact that perturbations of intestinal glutamine level have far-reaching consequences, including IBD. NLRX1 keeps several processes in balance, such as the proliferation, tight junction function, glutamine metabolism, pro-inflammatory cytokine production of epithelial cells in a sirtuin 1 (SIRT1)-dependent manner, and might prevent dysbiosis and subsequent inflammation as well. Using intestinal organoids, it was demonstrated that NLRX1 deficient epithelial cells have significantly increased glutamate dehydrogenase activity that leads to augmented proliferation, decreased tight junction function and lower expression of SIRT1. The results indicate that NLRX1 deficient mice use more glutamine, which shapes the bacterial flora of the gut in a way that is more beneficial for colitogenic bacteria [56].

**Figure 6:**
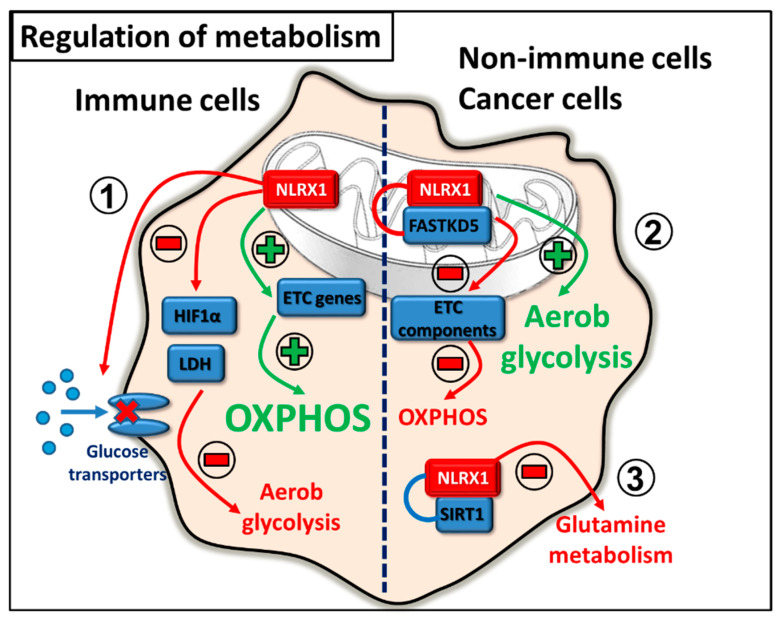
Regulation of metabolism by NLRX1. 1. In immune cells, NLRX1 blocks aerobic glycolysis through inhibiting the activity of HIF-1α and LDH, and glucose uptake, while inducing ETC gene expression to promote OXPHOS. 2. In non-immune cells or cancer cells, NLRX1 impairs the production of ETC components via interaction with FASTKD5, and subsequently attenuates OXPHOS, while supporting aerobic glycolysis. 3. NLRX1 negatively regulates glutamine metabolism in a SIRT1-dependent manner.

Furthermore, NLRX1 controls the amino acid level of the central nervous system (CNS) as well. In the CNS, extracellular neurotransmitter and glutamate levels must be carefully balanced to avoid excitotoxicity and neuronal death. Astrocytes act as a buffer by regulating extracellular glutamate levels in CNS, however; this function might be compromised upon inflammation. Mahmoud et al. demonstrated that NLRX1 is required for both glutamate uptake and inhibition of excess glutamate release by astrocytes. NLRX1 promotes ATP production to cover the energy needs of glutamate transporters, and inhibits Ca^2+^ release from the endoplasmic reticulum to suppress glutamate exocytosis [57] (Figure 6).

## 3. Immune Cell Related Actions of NLRX1

### 3.1. Regulation of Myeloid Cell Functions by NLRX1

#### 3.1.1. Macrophages

Macrophages represent a highly plastic and functionally heterogeneous group of cells, which play a role in the initiation as well as in the resolution of inflammation, thus are key cellular mediators of immune responses [58,59]. The local tissue microenvironment has been reported to greatly influence the polarization of macrophages [60,61]. Interestingly, the role of NLRX1 have been examined only in the classical inflammatory M1 type of macrophages, whereas no data are available regarding the anti-inflammatory M2 type of macrophages that could help to understand the complexity of NLRX1 driven regulation in plastic cell populations.

As key cells in the immune response to infectious agents, macrophages act mainly against bacteria and fungi, but are also implicated in antiviral responses as well. In most of the studied cell types, NLRX1 functions as a negative regulator of RLR-mediated type I IFN signaling by inhibiting interaction between RIG-I and MAVS. However, interestingly, three independent research groups using differentially generated NLRX1-KO mice observed that NLRX1 is not involved in regulation of MAVS-dependent type I IFN responses in macrophages. Allen et al. published that in C57BL/6 NLRX1-deficient mice with the mixed genetic background of IC1 C57BL/6 and Balb/c, poly (I:C) triggered IFNβ production of mouse BMDMs was not affected by NLRX1 deficiency. Furthermore, no difference was observed between the BMDMs from NLRX1-KO and wild type mice upon infection with *Legionella pneumophila* or *Listeria monocytogenes* bacteria, which are strong inducers of IFNβ secretion in macrophages [19]. Importantly, these results are in line with the observations of two other research groups. Rebsamen et al. also generated NLRX1-deficient mice from a C57BL/6-129/SvJ mixed background and observed normal MAVS-dependent antiviral responses in NLRX1-deficient BMDMs infected with Sendai virus [62]. Similar data were published by Soares et al. using C57BL/6 NLRX1-deficient mice generated from the crossing of floxed and Cre mice. The expression level of antiviral (IFNα/β, CXCL10, STAT2, IRF7) and inflammatory (IL-6, KC) genes did not differ between NLRX1-KO and wild type BMDMs following infection with Sendai virus [63]. Based on the data the authors concluded that NLRX1 does not interfere with the MAVS-dependent antiviral signaling in macrophages.

On the contrary, it seems that NLRX1 could be a potent regulator of the MAVS-independent NF-κB signaling in macrophages. Stimulation with LPS, which activates TLR4 in a MAVS-independent manner [64], resulted in significantly elevated IFNβ, IL-6 and IL-1β secretion by NLRX1-KO BMDMs compared to wild type cells. Mechanistically it was shown that NLRX1 directly interacted with TRAF6 and inhibited TLR4/MyD88-mediated NF-κB signaling in macrophages [19].

*Helicobacter pylori* (*H. pylori*) is a pathogenic Gram-negative bacterium, which can induce chronic infection of the human gastrointestinal tract that can eventually lead to the development of ulcer or gastric cancer (GC). In a study, involving Chinese individuals, NLRX1 was one of the 51 identified gene polymorphisms, which are associated with the increased risk of GC development during a *H. pylori* infection. PCR array analysis of NLR signaling-associated and inflammasome-related molecules revealed that *H. pylori* infection significantly downregulated NLRX1 expression and induced NF-κB signaling in THP-1 human monocyte-like cells. Based on these results the authors concluded that perturbation of NF-κB signaling due to NLRX1 gene polymorphism and/or its decreased expression following *H. pylori* infection makes individuals more susceptible to GC development [65].

In line with these results, significant and early downregulation of NLRX1 transcript was detected by another study using time course transcriptome analysis of BMDMs upon *H. pylori* infection. Using in silico modeling and simulation tools, the research group found that loss of NLRX1 leads to a prompt increase in NF-κB signaling resulting in higher levels of early cytokines but not of sustained or late cytokines. Experimental validation of computational modeling predictions demonstrated that NLRX1-KO BMDMs produced significantly more IFNγ and ROS compared to the wild type cells after infection [66].

Interestingly, the endogenous or pathogen-derived ligands of NLRX1 highly determine the regulatory function of NLRX1 and can modify the outcome of immune responses. Using the C-terminal region of NLRX1 as a putative ligand-binding domain, diverse compounds from three libraries were screened in an in silico molecular docking approach to identify novel endogenous ligands for NLRX1. This virtual screening identified different lipids such as punicic acid (PUA) and docosahexaenoic acid (DHA) as potential ligands for NLRX1, then binding of the molecules were experimentally verified using surface plasmon resonance spectroscopy. In vitro treatment of BMDM with PUA and DHA suppressed the LPS-activated NF-κB signaling, and the inhibitory effect of both components was abrogated in the NLRX1-KO BMDM. The anti-inflammatory effect of PUA was mediated through NLRX1, which was further confirmed in a DSS-induced colitis model, where PUA alleviated symptoms of wild type animals, but did not reduce colitis symptoms of NLRX1-KO mice. The virtual screening of a lipid library for further putative NLRX1 ligands also identified polyketides, prenol lipids, and plant sterol lipids, which possess anti-microbial, antioxidant or low-density lipoprotein (LDL) lowering effects, respectively, as well as CoA-containing fatty acids, which are important contributors of mitochondrial energy metabolism. These results suggested for the first time that NLRX1 can also act as a cytosolic lipid sensor and may function at the crossroad of metabolism and immune responses [67].

In a study to identify molecules involved in the DNA-induced innate immune response, another NLRX1 interacting protein phosphatase, the Eyes absent (EYA) 4 was identified as a regulator of IFNβ secretion in MEFs. EYA4 is a protein phosphatase with dual function that depends on its cellular localization. In the nucleus, it functions as a co-transcription factor and modulates chromatin structure via its N-terminal tyrosine-phosphatase activity. However, Okabe et al. found that in the cytoplasm, it regulates innate immune reactions in response to undigested DNA of apoptotic cells by modulating signal transduction pathways via its C-terminal region. Following transfection of HEK293T cells with hemagglutinin (HA)-tagged NLRX1 and flag tagged EYA4 direct protein-protein interaction were confirmed by co-immunoprecipitation. To delineate its function in an immunocompetent cell, retroviral delivery of EYA4 to fetal liver macrophages was performed, that resulted in significantly enhanced IFNβ expression upon poly (I:C) treatment. Furthermore, point mutations generated in the EYA4 significantly reduced the poly (I:C)-induced IRF3-mediated IFNβ response in macrophages, while not affected the interaction of EYA4 with MAVS. The mechanism behind the observed phenomenon is not fully understood and remained to be determined to date [68].

Besides the endogenous ligands, some pathogens are also able to provide interacting ligands for NLRX1. For example, compared to wild type mice, significantly higher viral loads and inflammation were detected in the lungs of Nlrx1^−/−^ mice following influenza A virus infection. This was accompanied with a delayed and severely impaired type I IFN response, while the number of the alveolar macrophages was not different from that of the wild type. Upon stimulation with the synthetic RIG-I agonists 5′pppRNA or poly (I:C), there was no difference in the IFNβ secretion between BMDMs from NLRX1-KO and wild type mice. On the contrary, in NLRX1-KO macrophages significantly lower IFNβ secretion, enhanced mitochondrial damage and highly elevated apoptosis were detected following influenza A virus infection when compared to their wild type counterparts. It has been described previously that in the course of influenza A virus infection, viral PB1-F2 protein triggers the disruption of mitochondrial membrane potential that ultimately leads to the apoptosis of infected host cells. Jaworska et al. showed that NLRX1 directly binds PB1-F2 impairing mitochondria-mediated early apoptosis, thus NLRX1 indirectly allows type I IFN secretion by macrophages [26].

In another study, a member of the FAS death-inducing signaling complex, the FAF1 was identified as a positive regulator of IFNβ signaling via a direct interaction of NLRX1. FAF1 deficient mice showed lower resistance to VSV infection and produced less IFNβ and IL-6 compared to the wild type mice. Similar results were obtained using FAF1-KO BMDMs following H1N1 influenza virus infection, while there was no difference in the cytokine production following HSV infection indicating that FAF1 positively regulates IFNβ response to RNA viruses, without affecting that to DNA viruses. Furthermore, downregulation of FAF1 in BMDMs, RAW264.7 macrophages and THP-1 cells resulted in reduced IFNβ and IL-6 secretion. At the molecular level it was shown that the binding site for FAF1 overlaps with the binding site for MAVS on NLRX1 suggesting that FAF1 and MAVS compete for NLRX1 binding [23].

Upon HIV or DNA virus infection, NLRX1 was found to interact with the cytosolic DNA sensor STING to negatively regulate the TBK1-dependent type I IFN responses. Consequently, due to the reduced STING-dependent TBK1 activation, increased virus replication was observed in NLRX1 deficient human monocyte-derived macrophages and THP-1 cells [24].

It was also shown that NLRX1 directly binds TUFM, which then binds to ATG5-ATG12 and ATG16L1 to promote autophagy in cells. Importantly, decreased autophagy was detected in NLRX1-KO peritoneal macrophages following VSV infection compared to wild type cells. Based on these data, it was suggested that NLRX1 is a negative regulator of antiviral immune responses, since NLRX1 deficiency reduced autophagy and enhanced IFNβ secretion and eventually led to a better control of VSV replication [32]. ATG5-ATG12 are important components not only of the canonical but also the non-canonical autophagy pathway as well. Several fungal infections were reported to induce the non-canonical LAP in macrophages to kill fungi and to control ongoing infection [69,70]. For example, LAP formation was described in macrophages infected with *Histoplasma capsulatum,* which is a pathogenic fungus that survives phagocytosis and replicates in macrophages by interfering lysosomal acidification of phagolysosomes. Following fungal infection of mouse peritoneal macrophages, it was found that NLRX1 bound to TUFM and induced ATG5-ATG12 complex formation and promoted LC3-II incorporation into the fungus containing phagosomes. Besides autophagy, NLRX1-TUFM complex positively affected cytokine production of macrophages, thus reduced MAPK pathway activity in NLRX1 deficient cells. Most importantly, NF-κB signaling pathway was not modified in NLRX1-KO macrophages following *Histoplasma capsulatum* infection [36].

During mitophagy, which is a modified form of autophagy, damaged or senescent mitochondria are eliminated by an autophagosome formed around the mitochondria. It was shown that the virulence factor (LLO) of intracellular *Listeria monocytogenes* bacteria induced mitophagy in mouse peritoneal macrophages to promote bacterial survival. This mitophagic process was mediated by direct binding of LC3 to NLRX1 through its LIR motif indicating that NLRX1 can also function as a mitophagy receptor. NLRX1 deficiency of mouse peritoneal macrophages completely abrogated Listeria-induced degradation of mitochondrial proteins and DNA, and increased the number of damaged mitochondria and the production of mtROS. Furthermore, in NLRX1-KO mouse macrophages the Listeria titer was lower compared to the wild type macrophages, suggesting that Listeria hijacks host NLRX1 dependent mitophagy to support its own survival. As a mechanism the authors identified LLO triggered conformational changes in NLRX1 that resulted in the release of LRR domain from the NACHT domain. This change unleashed NLRX1 from its repressed state and allowed oligomerization and subsequent binding of LC3 through its LIR domain to induce mitophagy. Interestingly, basal and starvation-induced autophagy was not altered in NLRX1-KO mouse peritoneal macrophages, and neither NF-κB signaling nor IFNβ response were influenced by the absence of NLRX1 [37].

Besides pathogen-induced immune responses, functions of NLRX1 in macrophages were also investigated in association with a variety of diseases and trauma. Multiple sclerosis (MS) is a T cell-mediated autoimmune disease characterized by the inflammation of the CNS. The inflammatory condition is mainly generated by resident brain macrophages (microglia) and infiltrating monocytes/macrophages that eventually leads to neuronal demyelination and axonal damage. In experimental autoimmune encephalomyelitis (EAE), which is a widely accepted murine model of MS, a higher expression of MHC class II molecule was detected from CD45low CD11b+ resident microglia cells of the spinal cord of NLRX1-KO mice compared to the wild type animals. Furthermore, following LPS/IFNγ treatment, elevated IL-6 and CCL2 secretion, and increased nitric oxide synthase (NOS) 2 and MHCII expression was detected in cultured microglia isolated from NLRX1-KO neonatal mice compared to wild type animals, indicating that NLRX1 attenuates microglia activation and potentially represses disease progression [71].

In a mouse model of urethane-induced tumorigenesis, NLRX1-KO mice developed histiocytic sarcoma in the spleen and increased number of macrophages were detected compared to wild type animals. In serum-free conditions, BMDMs from NLRX1-KO mice showed significantly increased proliferation and expression of CCL2 and granulocyte colony-stimulating factor (G-CSF), which are involved in macrophage recruitment and proliferation, respectively. In contrast to previous reports, the authors did not find differences in cell death between KO and wild type BMDMs that might be due to the serum-free conditions suggesting stimulation specific mechanisms. Interestingly, they found that NLRX1 mediates cell death in neoplastic but not in normal cells and suggest that NLRX1 mediates tumor suppression through the induction of apoptosis [31] (Table 1).

The above data indicate that numerous contradictory results were reported concerning the activity of NLRX1 in macrophages. Often, similar stimulations or infection models applied on macrophages with different origin resulted in diverse regulatory mechanisms. Therefore, there might be differences in the regulatory action of NLRX1 between bone marrow derived and peritoneal macrophages since their functions are adapted to divergent tissue environment. Furthermore, the results also clearly show that stimulation of similar type of macrophages with different pathogenic microbes might lead to opposing results since fungi, intracellular and extracellular bacteria, RNA and DNA viruses can activate various signaling pathways depending on the sensing of their PAMPs by the host. Besides, these pathogens can also use one or more evading strategies to avoid host immune defense that might highly influence the regulatory mechanisms of immune responses. Altogether, in macrophages, the regulatory feature of NLRX1 appears to vary depending on the experimental condition, the type of pathogen and the origin of macrophages as well.

#### 3.1.2. Dendritic Cells (DC)

As the most efficient professional antigen presenting cells (APC), DCs link innate and adaptive immunity by inducing the activation of naïve T cells and shaping their polarization according to the immune system’s needs. In addition, they are also essential in maintaining peripheral tolerance. Several DC subsets with distinct phenotypical and functional characteristics have been described so far, including conventional DC 1 (cDC1), cDC2, pDC, Langerhans cell and monocyte-derived DC (moDC) [72]. Fekete et al. analyzed the contribution of regulatory NLRs, namely NLRX1 and NLRC5, to the RLR-mediated cytokine responses of human moDCs and pDCs, as discussed below [12]. Fekete et al. reported for the first time that NLRX1 serves as an important negative regulatory molecule that prevents the excessive inflammatory response of human DCs upon viral infection. Silencing of NLRX1, which is constantly expressed in human pDCs, resulted in enhanced type I IFN secretion upon stimulation with synthetic RLR ligands or VSV. Interestingly, the RLR-mediated NF-kB activation and pro-inflammatory cytokine production was not affected by NLRX1 silencing. It was also found that NLRX1 did not influence the production of IL-6 and TNF pro-inflammatory cytokines and IL-8 chemokine in pDCs activated with ligands for TLR7, TLR9 and TLR1/2 indicating that the TLR-mediated NF-kB signaling is not controlled by NLRX1. This is in line with a previous study showing that NLRX1 deficiency does not affect the type I IFN production in mouse pDCs activated with TLR3, TLR7 or TLR9 agonists [19]. In contrast to pDCs, human monocytes exhibit no or low levels of NLRX1, but upregulate its expression during their differentiation into moDC. These results show that NLRX1 negatively regulates both the type I IFN and pro-inflammatory cytokine production of moDCs activated with synthetic RLR ligands or live VSV. Collectively, this work demonstrates that NLRX1 mostly controls the RLR-mediated innate immune responses of human DCs [12].

Studies using mouse BMDMs demonstrated that RNA virus infection induces interaction between FAF1 and NLRX1, which inhibits binding of MAVS to NLRX1, thereby enabling the activation of MAVS-RIG-I-mediated antiviral signaling cascade. Suppression of FAF1 lowers the poly (I:C)-, VSV-, and influenza virus-induced production of IFNβ and IL-6, while increases viral replication rate in mouse bone-marrow-derived DCs (BMDCs) suggesting decreased antiviral and inflammatory responses [23]. These data indicate that the regulatory effect of FAF1 on NLRX1 also works in DCs similar to its action in macrophages.

A very recent study published that besides regulating antiviral responses, NLRX1 plays a role in antifungal immunity as well. Kastelberg et al. investigated the effect of NLRX1 deficiency in different immunocompetent and immunosuppressed mouse models of invasive pulmonary aspergillosis [73]. Regardless of the immune status of mice, NLRX1 deficiency lead to enhanced pulmonary inflammation, elevated levels of infiltrating immune cells and increased fungal burden. The significantly higher rate of mortality in NLRX1 deficient animals was the consequence of increased numbers of recruited IL-4-producing CD103+ DCs, which promoted unfavorable Th2-mediated immune responses. The enhanced IL-4 producing capacity of NLRX1 deficient DCs was found to be a consequence of increased JNK/JunB pathway activation in response to Aspergillus infection [73] (Table 1).

All the previous findings suggest that NLRX1 functions mainly as an inhibitor of various immune signaling pathways in DCs, however further studies are needed to unravel its precise role and the underlying molecular mechanism of its action in different DC subsets.

#### 3.1.3. Other Myeloid Cells

Besides macrophages and DCs, granulocytes and monocytes also belong to the myeloid lineage of the hematopoietic system. As part of the innate immune system, these cells circulate in the blood and are recruited first to the sites of infection or tissue injury. Within the peripheral tissue they are rapidly activated and adopt different effector functions such as phagocytosis, cytokine secretion and oxidative burst to secure a potent immune response against the invading pathogens [54].

So far there is only one study in the literature that specifically investigates the functions of NLRX1 in granulocyte and monocyte populations. Kors et al. studied the role of NLRX1 in the host defense against *Escherichia coli* induced pyelonephritis in a mouse model of experimental urinary tract infection [74]. They observed that absence of NLRX1 results in decreased bacterial clearance, but has no impact on the number of circulating leukocytes or immune cells in the bladder of mice with pyelonephritis caused by *Escherichia coli*. In addition, it was revealed that NLRX1 does not influence the pro-inflammatory cytokine secretion upon ex vivo whole blood LPS stimulation, and has no effect on the phagocytic capacity of granulocytes and monocytes suggesting that the reduced bacterial clearance cannot be explained by impaired granulocyte or monocyte responses [74]. They concluded that NLRX1 can be involved in urinary tract infections, however, it does not affect directly the functions of granulocytes and monocytes.

In another study, the role of NLRX1 was investigated regarding the functions of cells bearing CD11b+ [75], which marker is highly expressed on the surface of monocytes, neutrophils and other granulocyte subpopulations. However, it is important to note that CD11b also presents on the surface of many other leukocytes including natural killer (NK) cells, macrophages, some spleen cells or bone marrow cells. Theus et al. investigated the role of NLRX1 in a murine model of traumatic brain injury [75]. Secondary brain injuries are characterized by active immune responses like infiltration of immune cells, neuroinflammation, cerebral edema, hypoxia or ischemia. Following brain injury, NLRX1 deficient mice displayed more severe motor dysfunctions and could be characterized with larger cortical lesion areas compared to wild type animals. In the brain lesions of NLRX1 deficient animals the genes of both NF-κB signaling and apoptosis were significantly upregulated. CD11b+ cells, which are basically represented by microglia in the intact brain and are further expanded by infiltration of neutrophils and macrophages upon blood brain barrier disruption, were identified as the main contributing source of enhanced NF-κB signaling in these lesions. Besides dampening NF-κB signaling in CD11b+ cells, NLRX1 was found to attenuate brain injury via limiting apoptosis in neuronal cells as well. Moreover, the expression of NLRX1 was significantly downregulated, while NF-κB signaling was upregulated in patients with ruptured aneurysm. All these data imply, that NLRX1 has a protective role against brain injury-induced inflammation and neuronal loss [75] (Table 1).

However, the role of NLRX1 is widely studied in DCs and macrophages, our knowledge is limited with regard to other myeloid cell population, especially to the granulocytes, which are also major components of inflammatory reactions. In addition, granulocytes consist of three subtypes with diverse functions and pathological roles [76]. For example, neutrophils contribute to various autoimmune diseases [77], whereas the basophils and eosinophils can be important elements of allergic reactions [78]. Therefore, further studies are needed to reveal the precise role of NLRX1 in less studied myeloid cell types and the role of NLRX1 in those diseases in which these cells are involved.

### 3.2. Regulation of Lymphoid Cell Functions by NLRX1

#### T Cells

T cells are highly specialized lymphocytes, which are core components of adaptive immunity via coordinating responses to pathogens, allergens, and tumors. Cytotoxic T cells kill transformed or virus infected human cells, helper T cells (Th) aid the activity of other immune cells and regulatory T cells (Treg) control inflammatory processes to avoid unwanted tissue injuries or autoimmune reactions. Upon antigen recognition, naive Th cells differentiate into different type of effector cells of which the most studied subtypes are Th1, Th2 and Th17 cells. Th1 cells coordinate immune responses to intracellular pathogens and are associated with pathology of several inflammatory diseases. Th2 cells protect the host from parasites; however, their uncontrolled activation can lead to the development of allergic responses. Th17 cells regulate immune responses against bacterial and fungal infection and have been implicated in the pathogenesis of various autoimmune diseases [79].

By regulating T cell differentiation and effector functions, NLRX1 has the potential to become a therapeutic target mainly in inflammatory diseases. Leber at al. investigated the role of NLRX1 in three different mouse models of IBD including cellular-, dextran sodium sulfate (DSS)-, and *Citrobacter rodentium*-induced colitis. NLRX1 deficiency resulted in enhanced disease pathology in DSS-induced colitis. Adoptive transfer of naive or effector NLRX1 deficient CD4+ T cells also enhanced disease severity. Similarly, in *Citrobacter rodentium*-induced colitis, T cell—specific deletion of NLRX1 resulted in greater inflammation. In the absence of NLRX1, increased expression of IL-17, IFNγ and TNFα were observed in the colon. In line with this, expansion of Th1 and Th17 populations was detected, whereas no difference in the Treg numbers were described within the colonic lamina propria and spleen. Mechanistically it was observed that in the absence of NLRX1, the intensified aerobic glycolysis promotes severe inflammatory phenotype and exacerbates disease symptoms [51].

Another study investigated the role of NLRX1 in IBD by the overactivation of the receptor. The NLRX1 activator, NX-13 is a promising therapeutic candidate for the treatment of IBD. Orally administered NX-13 decreased disease severity and pathology in three different mouse models of IBD proving the protective role of NLRX1. NX-13 administration reduced the numbers of Th1, Th17 cells and neutrophils, whereas increased the proportion of IL-10—producing Tregs in the colonic lamina propria. In vitro NX-13 decreased the differentiation of naïve CD4+ T cells into Th1 and Th17 cells implying their dependence on the presence of NLRX1. The beneficial effects of NX-13 are provided by the attenuation of inflammation supporting aerobic glycolysis [52].

Besides IBD, NLRX1 can also be a potential target to treat autoimmune MS. Gharagozloo et al. studied the role of NLRX1 in EAE, which is an animal model of autoimmune brain inflammation [80]. Interestingly, when myelin-specific T cell receptor transgenic mice (2D2) were crossed with NLRX1 deficient mice, the development of spontaneous EAE increased by 10 times compared to the 2D2 mice and lead to increased CNS tissue inflammation, indicating the importance of NLRX1 in preventing EAE onset. Although, the numbers of T cells and B cells were comparable in both genotypes, NLRX1 deficient CD4+ T cells showed greater proliferation rate and higher ability to differentiate into encephalitogenic Th1 and Th17 cells compared to 2D2 T cells. Moreover, increased myeloid cell activation was observed in the CNS of NLRX1 deficient mice, thus the authors speculate that NLRX1 acts on two levels to prevent initiation and progression of the disease. On the one hand, it inhibits the proliferation and differentiation of autoreactive T cells, and on the other hand it reduces the activation and infiltration of CD11b+ MHCII+ myeloid cells to the CNS [80].

A more recent research demonstrated that exogenous administration of NLRX1 could alleviate the severity of EAE [81]. A novel fusion protein was created by conjugating the LRR domain of NLRX1 with dNP2, a blood-brain barrier (BBB)-permeable peptide, which can deliver cargo to the CNS by penetrating the BBB via multiple endocytotic pathways [82]. It was found that dNP2 was able to efficiently deliver the LRR domain of NLRX1 into CD4+ T cells. Most importantly, intraperitoneal administration of the dNP2-LRR fusion protein attenuated disease progression probably via decreasing the numbers of infiltrating IFNγ producing CD4+ T cells and reducing demyelination in the spinal cord. Under in vitro conditions, the fusion protein significantly reduced the differentiation of naïve CD4+ T cells into IFNγ producing Th1 cells but not into Th17 or Treg cells suggesting that dNP2-LRR specifically regulates Th1 differentiation. Since the dNP2-LRR slightly reduced the expression of T-bet, the specific transcription factor of Th1 cells, the authors presumed that dNP2-LRR acts probably through interfering with the NF-κB signaling pathway. Based on previous reports, they also speculated that their observations might be related to NLRX1-mediated metabolic changes as well [81] (Table 2).

The above data suggest that NLRX1 has a potent regulatory role in the activation, differentiation and proliferation of CD4+ T cells suggesting its importance in T cell–mediated diseases. In overall, NLRX1 deficiency seems to worsen the severity of autoinflammatory or autoimmune diseases suggesting that a deeper understanding of mechanisms regulating NLRX1 actions might offer novel therapeutic strategies for the treatment of IBD, multiple sclerosis and other T cell -mediated diseases.

### 3.3. Regulation of Peripheral Blood Mononuclear Cell (PBMC) Function by NLRX1

Besides T cells, the functions of NLRX1 in other lymphoid immune cells, including B cells or NK cells, are not well characterized yet. Some of the previously mentioned studies often use PBMC derived from patients with inflammatory conditions to confirm their results obtained from mouse models. PBMC is a mixture of diverse immune cells, including T cells, B cells, NK cells, monocytes and different blood DC subtypes. In humans, the largest fraction of PBMC is represented by lymphocytes, which is followed by monocytes and a small percentage of DCs. Owing to their simple accessibility and easy phenotyping, PBMCs are useful tools in research and clinical studies. Nevertheless, it is worth to note, that due to the complexity of cell to cell interactions in PBMCs under in vitro experimental conditions, it is difficult to draw definite conclusions and make adequate predictions for the in vivo situation [83].

In the PBMCs of ulcerative colitis (UC) patients, the NLRX1 activating NX-13 drug candidate decreased the number of IFNγ, TNFα and IL-4 positive cells, while increased the number of IL-10 producing cells in a concentration-dependent manner. NX-13 also reduced NF-κB-mediated production of IL-6, IL-8 and monocyte chemoattractant protein 1 (MCP1) following stimulation with PMA and ionomycin, or TNF. In addition, NX-13 decreased ROS production upon treatment with hydrogen peroxide as well [52]. As we described above, the research group observed similar effects on CD4+ T cells in mouse models of IBD. These results suggest that NLRX1 could be a promising therapeutic target for alleviating inflammation in primary cells of patients suffering from inflammatory bowel diseases [52].

Besides the mouse model of EAE, the possible involvement of NLRX1 in autoimmune brain disease was also confirmed using human PBMC culture. In PBMCs of relapsing-remitting MS (RRMS) patients, NLRX1 levels are significantly higher compared to healthy controls. Interestingly, the expression of NLRX1 was higher in CD14+ myeloid cell compartment than in the CD3+ T cell population. The authors speculated that the intensifying inflammation during disease progression triggers NLRX1 upregulation as a possible negative feedback regulatory loop. In addition, 6 rare NLRX1 mutations were identified, including a p.Glu192Ter truncation mutation, which was found in 10 out of 24 MS patients, the harboring of which could increase the risk for developing MS [80].

Another study investigated the aggregate forming capacity of MAVS in the PBMCs of systemic lupus erythematosus (SLE) patients, the prion-like aggregation of which leads to enhanced type I IFN signaling that consequently fuels autoimmunity [84]. Interestingly, NLRX1 expression did not differ between MAVS aggregate positive and negative SLE patients. However, NLRX1 resides mainly in the mitochondria, its expression was also detected in the cytoplasmic fraction of PBMC preparations from SLE patients [84].

Regarding HIV infection, Nasi et al. compared NLRX1 mRNA levels in the PBMCs of healthy controls to those of HIV positive patients taking effective combined antiretroviral therapy (cART) [85]. Interestingly, HIV positive patients showed significantly reduced levels of NLRX1 than healthy controls. Nonetheless, there was not any difference in the expression of NLRX1 among CD14+ monocytes, CD4+ and CD8+ T cells and CD19+ B cells from either HIV positive patients or healthy individuals. Since NLRX1 protects against virus-induced apoptosis, the authors speculate that NLRX1 downregulation might serve as a viral escape mechanism via triggering cell death in various immune cell types [85] (Table 3).

Despite the fact that the cell specific activities of NLRX1 cannot be studied using PBMCs, experiments with them are highly important, since those provide valuable information about the association of NLRX1 functions with various human pathological conditions and thus might highlight the potential value of NLRX1 as a therapeutic target.

### 3.4. Regulation of Immune-Related Structural Cell Functions by NLRX1

#### Epithelial Cells and Fibroblasts

Tightly packed epithelial cells form a physical barrier by covering external surfaces and lining internal areas of the body and thus serve as our first line of immune defense against invading pathogens. Since they are equipped with a diverse array of PRRs, they are essential for mounting a protective immune response [86]. Fibroblasts and fibroblast like cells provide the framework for tissues by synthetizing collagen and extracellular matrix components and are critical in wound healing as well. Besides their canonical functions, pathogen sensing fibroblasts can synthetize antimicrobial peptides, cytokines, chemokines and growth factors, which are important to initiate inflammatory responses [87].

Epithelial cells ensure a thigh barrier at the possible entry sides of the pathogens and inhibit the invasion of hazardous microbes in the human body. In airway epithelial cells, NLRX1 was demonstrated to promote virus induced epithelial barrier disruption upon rhinovirus (RV) infection. Authors showed that after RV infection, NLRX1 translocated to mitochondria and facilitated the RV induced mtROS production, which contributed to tight-junction disruption [39].

In epithelial-like HEK293T and HeLa cells, NLRX1 acts as a negative regulator of RLR-mediated antiviral signaling, as it decreases type I IFN production following viral infection via interacting with the adaptor protein MAVS and inhibiting IRF3 dimerization [14]. On the contrary, another research group demonstrated that loss of NLRX1 had no effect on the type I IFN response of HEK293T cells in response to Sendai virus infection. The authors also highlighted the fact that overexpression of the NLR proteins such as NLRX1 and NLRC3, resulted in artifactual inhibition of Luciferase Reporter Assay, thus cannot be considered as a reliable method to study the inhibitory functions of overexpressed NLR proteins [88].

Upon fungal infection of mice by *Aspergillus fumigatus*, NLRX1 deficiency resulted in increased pulmonary inflammation and immune cell recruitment as a result of excess production of chemokines and cytokines by airway epithelial cells. Similarly, NLRX1 deficiency in human BEAS-2B airway epithelial cells enhanced chemokine and pro-inflammatory cytokine (CXCL8, CXCL1, and IL-6) production that was found to be mediated by elevated p38 phosphorylation in response to *Aspergillus* infection [73]. In contrast, the NLRX1 was described as a positive regulator in epithelial-like cells. In HEK293T cells, NLRX1 has been identified as an activator of ROS generation, since it increased the TNFα and *Shigella* infection-induced production of ROS thereby potentiating NF-κB-, and JNK-dependent pro-inflammatory signaling pathways [43].

Interestingly, NLRX1 was found to act as a double-edged sword in gingival epithelial cells upon infection with the oral commensal *Fusobacterium nucleatum*. On one hand, it triggers ATP-induced NLRP3 inflammasome activation via enhancing mtROS production, on the other hand it negatively regulates NF-κB-mediated IL-8 expression. Based on these results, the authors concluded that NLRX1 promotes the recognition of commensal bacteria, while decreasing the production of pro-inflammatory mediators to prevent excessive inflammation under normal conditions [89].

IBD patients display increased epithelial permeability, which allows bacterial translocation and exposure to gut microbial antigens resulting in the activation of immune cells and subsequent development of chronic intestinal inflammation [90]. Leber at al. described that NLRX1 deficient mice with DSS-induced colitis represent more severe inflammation and disease pathology with enhanced intestinal expression of neutrophil-attracting chemokines, pro-inflammatory cytokines and antimicrobial peptides [56]. Furthermore, NLRX1 deficient mice show decreased tight junction gene expression and increased intestinal permeability. Moreover, loss of NLRX1 in intestinal epithelial cells altered SIRT1-dependent metabolism and promoted cell proliferation implying potential harmful post-inflammatory effects such as cancer development. The modified epithelial cell behavior also affected the composition of the microbiome that was reflected in the increased proportion of inflammation-associated bacterial strains in NLRX1 deficient mice [56].

Studies often use MEFs or fibroblast-like cell lines to explore the exact molecular mechanisms behind the regulatory effects of NLRX1 observed on primary mouse macrophages or to corroborate the results obtained with macrophages. As we mentioned previously, Rebsamen et al. described that NLRX1-deficient BMDMs infected with Sendai virus displayed normal MAVS-dependent antiviral responses [62]. Likewise, they did not observe any changes in MAVS-dependent IRF3 phosphorylation and in the levels of IFN-β and IL-6 in response to poly (I:C) in primary NLRX1-deficient MEFs either [62].

Similarly, Soares et al. published that NLRX1-deficiency did not affect the expression of IFNβ, IL-6, KC and CXCL10 in MEFs infected with Sendai virus, Encephalomyocarditis virus (EMCV) or VSV. These results are in line with data obtained with NLRX1-KO BMDMs [63] suggesting that NLRX1 does not influence MAVS-dependent signaling in either MEFs or macrophages. However, in contrast to macrophages, discrepancies between published data on the role of NLRX1 in MEFs reflect that there is not a clear consensus about its exact function in MAVS-dependent responses yet. For instance, Allen et al. demonstrated normal IFNβ production in NLRX1 deficient MEFs following infection with EMCV, which is recognized by MDA5 and not RIG-I [91]. However, they observed elevated IFNβ and IL-6 production in NLRX1 deficient MEFs upon Simian virus 5 (SV5), Sendai virus, VSV and influenza A virus infections. Based on these data, they concluded that NLRX1 negatively mediates RIG-I/MAVS signaling, while does not affect MDA5/MAVS pathway in primary MEFs [19].

The same research group investigated the role of NLRX1 in LPS-triggered MAVS-independent NF-κB activation in MEFs generated from NLRX1 deficient mice as well. The lack of NLRX1 further enhanced the LPS-induced phosphorylation of p65 and decreased the levels of the NF-κB inhibitor IκBα compared to wild type cells. These results suggest that, similar to BMDMs, NLRX1 negatively regulates LPS-activated NF-κB signaling of MEFs via association with TRAF6 [19].

The interaction of NLRX1 with other cytosolic molecules was also approved in MEFs. Similar to fetal liver macrophages, EYA4 was also identified as a positive regulator of IRF3-mediated expression of IFNβ and CXCL10 in MEFs. EYA4 transfected MEFs displayed elevated IFNβ levels in response to *Escherichia coli*, mammalian DNA or poly (I:C) compared to non-transfected cells that was presumably mediated via the interaction of EYA4 and NLRX1 [68]. Moreover, upon VSV infection, NLRX1 and TUFM complex oppositely regulates autophagy and type I IFN production in MEFs, similar to mouse peritoneal macrophages. In NLRX1 KO MEFs, decreased LC3B-II levels were detected that led to defective autophagy. At the same time, the absence of NLRX1 was associated with increased IL-6, TNFα and type I IFN production. These findings reveal that NLRX1 through interacting with TUFM, ensures optimal level of autophagy, while decreases type I IFN production during viral infection [32].

Finally, in HeLa 229 cells and in MEFs, it was also confirmed that some bacteria are able to exploit the functions of NLRX1 to ensure their own survival. Upon *Chlamydia trachomatis* infection, it was found that NLRX1 contributes to *Chlamydia*-dependent ROS production, which is required to support chlamydial growth [40] (Table 4).

Similar to macrophages, epithelial cells and fibroblasts are extensively studied cells in the field of NLRX1 biology and the results further confirm that the role of NLRX1 might differ depending on the cell type and the applied stimuli or infection. It is important to mention that the exact mechanisms of NLRX1 regulatory functions are often investigated on epithelial or fibroblast like cells or cell lines [19,32], however the cell type specificity needs to be taken into account. Moreover, experiments with epithelial cells revealed that the opposing results could be also addressed to technical issues such as differences in overexpression and depletion techniques of NLRX1 [88], which might further increase the diversity of results. Thus, the discrepancies in the regulatory actions of NLRX1 are most likely multifactorial in origin.

## 4. Discussion

NLRX1, by regulating various biological functions, is implicated in the development of a wide variety of diseases. It controls host-pathogen interactions, autoinflammatory-, or autoimmune-related inflammations, metabolic diseases and it is also involved in oncological disorders. The multiple roles of NLRX1 in different pathological conditions have been already extensively reviewed in recent articles [17,18]. Interestingly, based on the published data, NLRX1 seems to exert different actions from condition to condition, and each of the previous reviews agree that the function of NLRX1 varies depending on the tissue environment and principally on the cell type. Therefore, in the present review, we aimed to characterize and classify the nature of NLRX1 mediated regulatory mechanisms in a cell type specific manner with a special focus on the immune cell-related actions of NLRX1.

Upon pathogenic infections, the tightly coordinated and overlapping effector functions of innate and adaptive cellular elements are required to recognize and eliminate invading pathogens as efficiently as possible. Potential entry sites for pathogens are mainly the peripheral tissues and mucosal surfaces, where the first line of defense is provided by the tight barrier-forming tissue epithelial cells and tissue-resident innate immune cells [92]. First, innate immune cells, such as tissue/organ resident macrophages and DCs interact with invading pathogens by recognizing them through their cell surface or intracellular PRRs, the activation of which leads to the initiation of downstream signaling pathways and subsequent antimicrobial responses. NLRX1 is able to interact with components of both TLR-, and RLR-mediated signaling pathways and thereby regulates the cellular processes associated with these receptors. NLRX1 is mainly described as a negative regulator of MAVS-dependent antiviral responses through its direct interaction with the MAVS adapter protein. Nevertheless, data suggest that NLRX1 does not influence the virus-initiated MAVS-dependent type I IFN production in macrophages [19,62,63], whereas it is a negative regulator in both pDCs and moDCs [12,19]. Much less data are available regarding the effects of NLRX1 on type I IFN production of epithelial cells. Interestingly, two studies using the same cell line, HEK293T cells and the same viral stimulation with Sendai virus, yielded different results. One group demonstrated no effect [88], whereas the other suggested a negative regulatory role for NLRX1 in MAVS-dependent type I IFN production [14]. However, studies performed on MEFs reveal that the type of virus and receptors involved in virus recognition may also influence the regulatory nature of NLRX1. Some research groups found no changes [62,63], whereas others published a negative regulatory role of NLRX1 in MAVS-dependent type I IFN production of MEFs as a response to viral infection [32]. The latter finding is also supported by a subsequent study showing that NLRX1 interferes with the MAVS pathway in response to RIG-I-, but not to MDA5-dependent viruses [19]. However, it must be noted that multiple viral sensors, such as endosomal TLRs or cytosolic receptors other than RLRs, contribute to the simultaneous detection of live viruses. Thus, in in vivo experiments, it is difficult to determine, which receptor is specifically regulated by NLRX1 that might contribute to the observed differences in the published data. Nonetheless, it is also important to emphasize that animal models provide a much more realistic representation of in vivo situations and allow the investigation of NLRX1 actions in more complex settings as compared to in vitro or ex vivo experiments. In addition, most in vitro studies investigating NLRX1-MAVS interactions use mostly the non-specific dsRNA analog, poly (I:C) for RLR activation. However, it is mainly sensed by MDA5, it can also be recognized by RIG-I [93], as well as by the endosomal TLR3 receptor when added endogenously or exogenously [94]. Thus, besides the cell type, the type of virus and viral sensor might also contribute to the generation of contradictory results regarding the features of NLRX1-mediated regulation.

Nevertheless, the regulatory role of NLRX1 in NF-κB-mediated pro-inflammatory responses is much more consistent and clear. Regardless of the type of stimuli, most articles suggest a negative regulatory role for NLRX1 in the NF-κB signaling of macrophages, DCs, epithelial cells, and MEFs that is principally mediated by interaction with TRAF6. On the contrary, pDC seems to be an exception among innate immune cells. Allen et al. using mouse pDCs [19] and our group using human pDCs [12] demonstrated that neither the RLR- nor the TLR-dependent NF-κB activation is affected by NLRX1 in pDCs. These observations suggest that the regulatory effects of NLRX1 are especially pronounced on those cellular functions, which are extensively used during the effector responses of the given cell. By producing many fold higher amounts of type I IFN than any other cell type, pDCs are most well-known for their antiviral activity [95]. On the contrary, their contribution to the secretion of NF-κB-mediated pro-inflammatory cytokines is negligible compared to macrophages or conventional DCs. Thus, it seems to be logical, that NLRX1 is an important regulator of the NF-κB-dependent antibacterial and antifungal responses of macrophages, which have high capacity to produce pro-inflammatory cytokines, whereas it functions primarily as a regulator of type I IFN responses in pDCs specialized for the production of type I IFNs. The special regulatory mechanism by NLRX1 is likely to play an important role in fine-tuning signals that mediate cytokine production to avoid an overactivated immune response and consequent tissue damages during inflammation.

In addition, it is important to note that many viruses have the ability to affect NLRX1 functions to facilitate their own invasion in the human body that might also contribute to the controversial data on the regulatory functions of NLRX1. In some cases, NLRX1 is hijacked by viruses via interaction with virus-derived molecules that has a pathophysiological importance. For example, in macrophages, NLRX1 positively regulates the antiviral immune response by sequestering PB1-F2, a small apoptosis-inducing protein of influenza A virus [26]. Moreover, SARS-CoV2 was found to target NLRX1 through its membrane-associated protein ORF9c that might lead to the inhibition of MAVS-mediated signaling [22]. Upon HIV-1 or DNA virus (HSV, vaccinia virus) infection, NLRX1 disrupts STING-TBK1 signaling, which mediates IFN production, and thus facilitates viral replication in myeloid cells [24]. Furthermore, rhinovirus promotes virus-induced epithelial barrier disruption via NLRX1 [39], whereas SIV upregulates NLRX1 expression in the early phase of the infection to potentiate viral replication [21].

Upon bacterial or fungal infection of myeloid cells, the ROS and autophagy modulating abilities of NLRX1 are predominated. In human epithelial cells, NLRX1 promotes *Shigella* induced ROS production, which potentiates important pro-inflammatory pathways, such as NF-κB and JNK signaling [43]. Similarly, upon *Fusobacterium nucleatum* infection of gingival epithelial cells NLRX1 activates the NLRP3 inflammasome via ROS, while negatively regulates NF-κB signaling and IL-8 expression [89]. Upon *Histoplasma capsulatum* infection NLRX1 promotes LAP and pro-inflammatory cytokine production in macrophages [36].

Besides viruses, bacteria can also make use of the multifunctional properties of NLRX1 to facilitate invasion of host cells. For example, in HeLa cells, *Chlamydia trachomatis* use NLRX1 to induce ROS production, which is essential for the survival of the pathogen [40]. In contrast, *Listeria monocytogenes* needs low intracellular ROS concentration for its survival, therefore it induces mitophagy via NLRX1 to decrease the number of mitochondria leading to reduced ROS production [37].

Upon sterile inflammation caused by environmental factors, autoimmune reactions or metabolic perturbation, NLRX1 is consistently referred to as an anti-inflammatory molecule, which aid to prevent undesirable inflammation through its negative regulatory effects on inflammatory cascades. By controlling T cell differentiation and effector functions through metabolic interventions, NLRX1 is a promising therapeutic target in inflammatory diseases. In different models of IBD the absence of NLRX1 drives uncontrolled T cell proliferation and leads to abnormal effector T cell functions via inducing a metabolic switch to aerobic glycolysis. Glycolysis favors the development of colitogenic effector T cell subpopulations, such as Th17 cell, which are characterized by increased proliferation capacity and decreased responsiveness to checkpoint inhibitors [51]. NX-13, the first NLRX1 targeting and activating drug was created to offer a novel method to treat inflammatory conditions [96]. Via inhibiting glycolysis, NX-13 impairs Th1 and Th17 differentiation and therefore alleviates IBD symptoms [52]. The immunometabolic changes of Th cells in EAE seem to be similar to the ones observed in IBD. Although the authors provided no data about the metabolic profile of NLRX1 deficient T cells in EAE, they speculated that the increased rate of proliferation and greater numbers of encephalitogenic Th1 and Th17 cells are presumably the result of enhanced glycolysis caused by the lack of NLRX1 [80]. In line with that, a BBB penetrating fusion protein consisting the LRR domain of NLRX1 (dNP2-LRR) impairs the activation of naive T cells and decreases the rate of infiltrating Th1 cells in inflamed brain tissues and alleviates EAE symptoms [81]. Based on these data it seems that in immune cells, especially in T cells, NLRX1 negatively regulates glycolysis and presumably favors OXPHOS, which rather promotes anti-inflammatory responses. Though, it is noteworthy, that opposing data have been published in cancer cells and non-immune cells. Cancer cells exhibit much higher glycolytic rate compared to normal cells, which under normal conditions utilize OXPHOS and switch to glycolysis only following activation to cover their energy needs [97]. Interestingly, inhibition of glycolysis lowered NLRX1 expression in cancer cells [53]. Moreover, NLRX1 was found to impair mitochondrial ETC activity [46] and inhibit the maturation of precursor transcripts for ETC complexes [55] in cancer cells to attenuate OXPHOS and facilitate aerobic glycolysis. These findings indicate that besides the cell type specificity, the metabolic profile of the cells might also influence the nature of NLRX1 actions. Contrary to previous data, which characterized NLRX1 as a tumor suppressor by inhibiting tumor promoting signaling pathways such as Akt, NF-κB or MAPK, it was also reported that NLRX1 contributes to tumorigenesis by regulating cell death. Since the role of NLRX1 in the regulation of tumor cell functions has been extensively reviewed elsewhere [18], we do not detail further in this paper. Overall, via regulating metabolic events, NLRX1 can influence the polarization of adaptive immune responses and thus might be a potent target to modulate T cell functions in T cell-associated inflammatory diseases.

The protective effect of NLRX1 in inflammation-associated diseases is also supported by a number of literature data. For example, NLRX1 alleviates hypoxia-induced apoptosis and inflammation during acute myocardial infarction [98], or acts as an anti-inflammatory agent in cerebral ischemia reperfusion injury via decreasing pro-inflammatory cytokine production [99]. Furthermore, NLRX1 deficiency leads to exacerbated LPS-induced heart injury by enhancing NF-κB and NLRP3 inflammasome activation [100]. Moreover, chronic obstructive pulmonary disease (COPD) patients show decreased expression of NLRX1, the suppression of which resulted in severe inflammation, alveolar remodeling, increased protease activity, apoptosis and inflammasome activation that have been shown to correlate with disease severity, worse prognosis and reduced quality of life [101]. Finally, an NLRX1 polymorphism (rs4245191), which predisposes patients to macrovascular complications and diabetic cerebral infarction, was identified as a risk factor for vascular complications of type 2 diabetes mellitus in the southern Han Chinese population [102].

The abovementioned studies and further examples have already been nicely reviewed in a previous publication [17]. Most of the clinical studies do not reveal details about the cell type specific regulation of NLRX1; however, these studies have a very important role in confirming the in vivo regulatory functions of NLRX1, which have been addressed only in in vitro or ex vivo models so far.

## 5. Conclusions

Based on the extensive and diverse literature data, it is not deniable that NLRX1 acting as a Swiss army knife is one of the most multifunctional receptor of mammalian cells. The secret behind its versatility is the ability to bind to many different intracellular components, thus NLRX1 can influence the outcome of various cellular processes. At the same time, we wish to emphasize that NLRX1 affects basic cellular functions, such as cytokine secretion, mtROS production, metabolism, autophagy or cell death, which actions can be attributed to almost every cell types. However, these processes are not used to the same extent by different cell types since each cell type is highly specialized to fulfill designated tasks. So far it seems that the major function of NLRX1 is to ensure protective, negative feedback mechanisms to prevent overzealous inflammatory reactions by inhibiting those cellular functions, which might drastically perturb the homeostasis of a given cell type. This cell type specificity might be identified as a hallmark of NLRX1 regulatory functions. It is also worth to mention that several endogenous or pathogen-derived exogenous factors might influence the outcome of NLRX1-mediated regulation, thus further studies are warranted to identify novel interaction partners of NLRX1 that might expand our knowledge of its functional heterogeneity. In order to better understand the molecular background of the complexity of NLRX1 regulatory mechanisms, in the future more comprehensive research is needed, which takes into account the cell specific aspect of NLRX1-mediated regulation and performs comparative analysis of multiple cell types simultaneously.

## Figures and Tables

**Table 1:** Regulation of myeloid cell functions by NLRX1.

I. Regulation of Myeloid Cell Functions by NLRX1				
Macrophages				
Cell Type	Model	Observed Effects	Mechanism	Ref.
NLRX1^−/−^ mouse BMDM	poly (I:C) challenge, *Legionella pneumophila* or *Listeria monocytogenes* infection	NLRX1 deficiency has no effect on IFNβ secretion	-	[19]
NLRX1^−/−^ mouse BMDM	Sendai virus infection	NLRX1 deficiency has no effect on IFNβ gene expression mediated by MAVS dependent signaling	-	[62]
NLRX1^−/−^ mouse BMDM	Sendai virus infection	NLRX1 deficiency has no effect on IFNα/β, CXCL10, STAT2, IRF7, IL-6 and KC gene expression mediated by MAVS dependent signaling	-	[63]
NLRX1^−/−^ mouse BMDM	LPS treatment	NLRX1 deficiency increases IFNβ, IL-6 and IL-1β production mediated by NF-kB dependent signaling	NLRX1 binds to TRAF6 and inhibits NF-kB signaling (demonstrated on MEFs)	[19]
human THP-1	*Helicobacter pylori* infection	Downregulation of NLRX1 expression upon infection, which is associated with enhanced NF-κB signaling	NLRX1 interferes with NF-κB pathway	[65]
mouse BMDM (in silico)	*Helicobacter pylori* infection	Infection induced downregulation of NLRX1 is associated with increased NF-κB signaling	NLRX1 interferes with NF-κB pathway	[66]
NLRX1^−/−^ mouse BMDM	*Helicobacter pylori* infection	NLRX1 deficiency increases IFNγ and ROS production	NLRX1 interferes with NF-κB pathway	[66]
mouse BMDM	PUA or DHA treatments together with LPS	PUA and DHA suppresses the LPS-activated NF-κB signaling	PUA or DHA binds to NLRX1 and inhibits NF-κB signaling	[67]
mouse fetal liver macrophages	EYA4 overexpression and poly(I:C) challenge	Overexpression of EYA4 enhances IFNβ expression	EYA4 binds to NLRX1 and enhances IRF3 signaling	[68]
NLRX1^−/−^ mouse BMDM	Influenza A virus infection	NLRX1 deficiency lowers IFNβ secretion	NLRX1 binds to viral PB1-F2	[26]
NLRX1^−/−^ mouse BMDM	Influenza A virus infection	NLRX1 deficiency enhances mitochondrial damages and apoptosis	NLRX1 binds to viral PB1-F2	[26]
FAF1^−/−^ mouse BMDM, mouse RAW264.7, human THP-1	VSV or H1N1 infections	FAF1 deficiency decreases IFNβ and IL-6 secretion and increases virus replication	FAF1 binds to NLRX1 and competes with MAVS	[23]
NLRX1^−/−^ human monocytes derived macrophages, THP-1	HIV or DNA virus infection	NLRX1 deficiency increases STING dependent type I IFN responses	NLRX1 binds to STING and inhibits STING dependent TBK1 activation	[24]
NLRX1^−/−^ mouse peritoneal macrophages	VSV infection	NLRX1 deficiency enhances IFNβ secretion	NLRX1 binds to TUFM/ATG5-ATG12 complex (demonstrated on MEFs)	[32]
NLRX1^−/−^ mouse peritoneal macrophages	VSV infection	NLRX1 deficiency reduces autophagy	NLRX1 binds to TUFM/ATG5-ATG12 complex (demonstrated on MEFs)	[32]
primary mouse peritoneal macrophages	*Histoplasma capsulatum* infection	Infection induces LC3-associated phagocytosis (LAP) via NLRX1	NLRX1 binds to TUFM/ATG5-ATG12 complex and promotes LC3-II incorporation into the phagosomes	[36]
NLRX1^−/−^ primary mouse peritoneal macrophages	*Histoplasma capsulatum* infection	NLRX1 deficiency decreases MAPK pathway dependent cytokine production	NLRX1 positively regulates MAPK pathway	[36]
NLRX1^−/−^ primary mouse peritoneal macrophages	*Histoplasma capsulatum* infection	NLRX1 deficiency has no effect on NF-κB signaling	-	[36]
NLRX1^−/−^ mouse peritoneal macrophages	*Listeria monocytogenes* infection	NLRX1 deficiency reduces mitophagy and inhibits Listeria growth	LLO of Listeria induces NLRX1 oligomerization and LC3 binding via the LIR domain of NLRX1	[37]
NLRX1^−/−^ mouse peritoneal macrophages	*Listeria monocytogenes* infection	NLRX1 deficiency increases mtROS production	undefined	[37]
NLRX1^−/−^ mouse peritoneal macrophages	*Listeria monocytogenes* infection	NLRX1 deficiency has no effect on NF-kB signaling and IFNβ response	-	[37]
NLRX1^−/−^ mouse microglia of spinal cord	EAE	NLRX1 deficiency elevates MHC II expression	undefined	[71]
NLRX1^−/−^ microglia from neonatal mice	LPS/IFNγ treatment	NLRX1 deficiency elevates IL-6, CCL2 secretion, and increases NOS2 and MHCII expression	undefined	[71]
NLRX1^−/−^ mouse BMDM	mouse model of urethane-induced tumorigenesis	NLRX1 deficiency increases CCL2 and G-CSF expression	undefined	[31]
**Dendritic Cells**				
**Cell Type**	**Model**	**Observed Effects**	**Mechanism**	**Ref.**
NLRX1 silenced human pDC	5′ppp-dsRNA or poly (I:C) treatment, VSV infection	Silencing of NLRX1 enhances type I IFN secretion	NLRX1 interferes with RLR-mediated antiviral signaling	[12]
NLRX1 silenced human pDC	5′ppp-dsRNA or poly (I:C) treatment, VSV infection,	Silencing of NLRX1 has no effect on pro-inflammatory cytokine production induced by RLR-mediated NF-κB activation	-	[12]
NLRX1 silenced human pDC	CpG-B, Imiquimod and PAM3CSK4 treatments	Silencing of NLRX1 has no effect on pro-inflammatory cytokine production induced by TLR-mediated NF-κB activation	-	[12]
NLRX1 silenced human moDC	5′ppp-dsRNA or poly (I:C) treatment, VSV infection	Silencing of NLRX1 enhances type I IFN secretion	NLRX1 interferes with RLR-mediated antiviral signaling	[12]
NLRX1 silenced human moDC	5′ppp-dsRNA or poly (I:C) treatment, VSV infection	Silencing of NLRX1 increases inflammatory cytokine production induced by RLR-mediated NF-κB activation	NLRX1 interferes with RLR-mediated NF-κB signaling	[12]
NLRX1^−/−^ mouse pDC	R848, CpG and poly (I:C) treatments	NLRX1 deficiency has no effect on TLR-mediated pathways	-	[19]
FAF1^−/−^ mouse BMDC	poly (I:C) treatment, *VSV and influenza virus infections*	FAF1 deficiency decreases IFNβ and IL-6 secretion and increases virus replication	FAF1 binds to NLRX1 and competes with MAVS	[23]
NLRX1^−/−^ mouse CD103+ DC	mouse models of invasive pulmonary aspergillosis	NLRX1 deficiency increases the promotion of IL-4-producing DCs and Th2 responses	NLRX1 interferes with JNK/JunB signaling pathway	[73]
**Other Myeloid Cells**				
**Cell Type**	**Model**	**Observed Effects**	**Mechanism**	**Ref.**
NLRX1^−/−^ mouse granulocytes, monocytes	*Escherichia coli* induced pyelonephritis	NLRX1 deficiency has no effects	-	[74]
NLRX1^−/−^ mouse CD11b + cells	murine model of traumatic brain injury	NLRX1 deficiency results in larger cortical lesions, more infiltrating cells and upregulated NF-κB activity	NLRX1 interferes with NF-κB signaling	[75]
NLRX1^−/−^ mouse CD11b+ cells	murine model of traumatic brain injury	NLRX1 deficiency enhances apoptosis	NLRX1 interferes with apoptotic pathway	[75]

**Table 2:** Regulation of lymphoid cell functions by NLRX1.

II. Regulation of Lymphoid Cell Functions by NLRX1				
T Cells				
Cell Type	Model	Observed Effects	Mechanism	Ref.
NLRX1^−/−^ mouse CD4+ T cells	Cellular-, DSS-, and *Citrobacter rodentium*-induced IBD	NLRX1 deficiency enhances the proliferation and differentiation of Th1 and Th17 cells	NLRX1 deficiency promotes metabolic shift towards aerobic glycolysis	[51]
mouse CD4+ T cells	DSS-induced IBD, administration of the NLRX1 activator, NX-13	NLRX1 activation decreases the differentiation of naïve CD4+ T cells into Th1 and Th17 cells	NLRX1 attenuates aerobic glycolysis and promotes OXPHOS	[52]
NLRX1^−/−^ mouse CD4+ T cells	*EAE* *(NLRX1 deficient* myelin-specific TCR transgenic 2D2 mice)	NLRX1 deficiency promotes the differentiation of autoreactive Th1 and Th17 cells	NLRX inhibits the differentiation of autoreactive T-cells and the activation and migration of myeloid cells	[80]
mouse CD4+ T cells	EAE, administration of the dNP2-LRR fusion protein	Administration of NLRX1 fusion protein inhibits T cell activation or Th1 cell differentiation	NLRX1 interferes with NF-κB signaling via reducing the level of T-bet	[81]

**Table 3:** Regulation of peripheral blood mononuclear cell (PBMC) function by NLRX1.

III. Regulation of Peripheral Blood Mononuclear Cell (PBMC) Functions by NLRX1				
Cell Type	Model	Observed Effects	Mechanism	Ref.
human PBMC	ulcerative colitis (UC), administration of the NLRX1 activator, NX-13	NLRX1 activation decreases the number of IFNγ, TNFα and IL-4 positive cells and reduces pro-inflammatory cytokine and chemokine levels	NLRX1 interferes with NF-κB signaling	[52]
human PBMC	ulcerative colitis (UC), administration of the NLRX1 activator, NX-13	NLRX1 activation decreases H2O2 triggered ROS production	undefined	[52]
human PBMC	relapsing-remitting MS (RRMS) disease	Increased NLRX1 expression is observed	Increased NLRX1 may serve as a negative feedback regulatory loop	[80]
human PBMC	*SLE disease*	No difference in NLRX1 expression between MAVS aggregate positive and negative patients	-	[84]
human PBMC	*HIV infection*	Decreased NLRX1 expression, which presumably serves as a viral escape mechanism	undefined	[85]

**Table 4:** Regulation of immune-related structural cell functions by NLRX1.

IV. Regulation of Immune-Related Structural Cell Functions by NLRX1				
Epithelial Cells and Fibroblasts				
Cell Type	Model	Observed Effects	Mechanism	Ref.
NLRX1 silenced human airway epithelial cells	rhinovirus infection, poly (I:C) treatment	Silencing of NLRX1 abrogates virus induced epithelial barrier disruption and decreased ROS production	NLRX1 promotes mtROS production	[39]
NLRX1 silenced human HEK293T, HeLa	Sendai virus infection	Silencing of NLRX1 promotes type I IFN production and decreases viral replication	NLRX1 interferes with MAVS signaling and inhibits IRF3 dimer formation	[14]
NLRX1 silenced human HEK293T	Sendai virus infection	Silencing of NLRX1 has no effect on type I IFN response	-	[88]
NLRX1^−/−^ mouse airway epithelial cells	mouse models of invasive pulmonary aspergillosis	NLRX1 deficiency enhances pulmonary inflammation and increases chemokine and cytokine production	NLRX1 impairs P38 phosphorylation	[73]
NLRX1^−/−^ human BEAS-2B airway epithelial cells	*Aspergillus fumigatus* infection	NLRX1 deficiency enhances chemokine (CXCL8, CXCL1) and cytokine (IL-6) production	NLRX1 impairs P38 phosphorylation	[73]
human HEK293T	Overexpressed NLRX1, *Shigella* infection, TNFα treatment	Overexpression of NLRX1 facilitates NF-κB and JNK pathways	NLRX1 promotes ROS production	[43]
NLRX1 silenced human gingival epithelial cells	*Fusobacterium nucleatum* infection	Silencing of NLRX1 attenuates the NLRP3 inflammasome activity	NLRX1 promotes mtROS production	[89]
NLRX1 silenced human gingival epithelial cells	*Fusobacterium nucleatum* infection	Silencing of NLRX1 increases NF-κB activity and enhances IL-8 production	NLRX1 interferes with NF-κB pathway	[89]
NLRX1^−/−^ mouse intestinal epithelial cells	DSS-induced colitis	NLRX1 deficiency increases proliferation, glutamine metabolism and pro-inflammatory cytokine production	NLRX1 interacts with SIRT1 and regulates glutamine metabolism	[56]
NLRX1^−/−^ mouse MEFs	poly (I:C) stimulation	NLRX1 deficiency has no effect on MAVS-dependent IFNβ and IL-6 production	-	[62]
NLRX1^−/−^ mouse MEFs	Sendai virus, EMCV and VSV infections	NLRX1 deficiency has no effect on MAVS-dependent IL-6, CXCL10, KC and IFNβ expression	-	[63]
NLRX1^−/−^ mouse MEFs	EMCV infection	NLRX1 deficiency has no effect on EMCV induced IFN-β production mediated by MDA5 activation	-	[19]
NLRX1^−/−^ mouse MEFs	Simian Virus, Sendai Virus, VSV and Influenza A virus infections	NLRX1 deficiency increases IFNβ and IL-6 production mediated by RIG-I activation	NLRX1 interferes with the RIG-I dependent MAVS signaling pathway	[19]
NLRX1^−/−^ mouse MEFs	LPS treatment	NLRX1 deficiency enhances p65 phosphorylation and decreases IκBα level	NLRX1 binds to TRAF6 and interferes with NF-κB signaling	[19]
mouse MEFs	EYA4 overexpression, *Escherichia coli* infection, mammalian DNA or poly (I:C) treatments	Overexpression of EYA4 enhances IFNβ expression	EYA4 binds to NLRX1 and enhances IRF3 signaling	[68]
NLRX1^−/−^ mouse MEFs	VSV infection	NLRX1 deficiency increases IL-6, TNF-α and type I IFN production	NLRX1 binds to TUFM/ATG5-ATG12 complex	[32]
NLRX1^−/−^ mouse MEFs	VSV infection	NLRX1 deficiency decreases LC3B-II level which leads to defective autophagy	NLRX1 binds to TUFM/ATG5-ATG12 complex	[32]
NLRX1 silenced human HeLa cells, NLRX1^−/−^ mouse MEFs	*Chlamydia trachomatis* infection	NLRX1 deficiency decreases the survival of the pathogen and the production of ROS	NLRX1 promotes ROS production and caspase-1 activation	[40]

## Abbreviations

APC	antigen presenting cell
ATG	autophagy-related protein
BBB	blood-brain barrier
BIR	baculoviral inhibition of apoptosis protein repeat
BMDCs	bone-marrow derived DCs
BMDM	bone marrow-derived macrophage
CARD	caspase recruitment and activation domain
cART	combined antiretroviral therapy
cDC	conventional DC
CIITA	Class II Major Histocompatibility Complex Transactivator
CLR	C-type lectin receptor
CNS	central nervous system
DAMP	damage-associated molecular pattern
DCs	dendritic cells
DHA	docosahexaenoic acid
DRP1	dynamin-related protein 1
DSS	dextran sodium sulfate
EAE	experimental autoimmune encephalomyelitis
EMCV	Encephalomyocarditis virus
ERK	extracellular signal-regulated kinase
ETC	electron transport chain
EYA	Eyes absent
FAF1	FAS-associated factor-1
FASTKD5	Fas-activated serine-threonine kinase family protein-5
GAS	Group A Streptococcus
GC	gastric cancer
G-CSF	granulocyte colony-stimulating factor
HCV	hepatitis C virus
HIF-1α	hypoxia-inducible factor 1-alpha
HIV	human immunodeficiency virus
HPV16	human papillomavirus 16
IBD	inflammatory bowel disease
IFN	interferon
IRF	IFN regulatory factor
JNK	c-Jun N-terminal kinase
LAP	LC3-associated phagocytosis
LC3	microtubule-associated protein 1 light chain 3
LDH	lactate dehydrogenase
LDL	low-density lipoprotein
LIR	LC3-interacting region
LLO	listeriolysin O
LPS	lipopolysaccharide
LRR	leucine-rich repeat
MAPK	mitogen-activated protein kinase
MAVS	mitochondrial antiviral signaling protein
MCP1	monocyte chemoattractant protein 1
MDA5	melanoma differentiation-associated protein 5,
MEF	mouse embryonic fibroblast
moDC	monocyte-derived DC
MS	multiple sclerosis
mtROS	mitochondrial reactive oxygen species
MTS	mitochondrial targeting sequence
NEMO	NF-kappa-B essential modulator
NF-κB	nuclear factor-kappa B
NK	natural killer
NLR	NOD-like receptors
NOD	nucleotide-binding oligomerization domain
OXPHOS	oxidative phosphorylation
PAMP	pathogen-associated molecular patterns
PB1-F2	polymerase basic protein 1-frame 2
PBMC	peripheral blood mononuclear cell
PCBP2	poly(rC) binding protein 2
pDC	plasmacytoid DCs
PKR	protein kinase R
poly (I:C)	polyinosinic:polycytidylic acid
PRR	pattern recognition receptor
PUA	punicic acid
PYD	pyrin domain
RIG-1	retinoic acid-inducible gene-1
RIP2	receptor-interacting-serine/threonine-protein kinase 2
RLR	RIG-I-like receptor
RRMS	relapsing-remitting MS
RV	rhinovirus
SARM1	Sterile Alpha and Toll/interleukin-1 receptor (TIR) Motif Containing 1
SIRT1	sirtuin 1
SIV	simian immunodeficiency virus
SLE	systemic lupus erythematosus
STING	stimulator of interferon genes
SV5	Simian virus 5
TA	acidic transactivation domain
TBK1	TANK-binding kinase 1
Th	helper T cells
TIR	Toll/interleukin-1 receptor
TLR	Toll like receptor
TRAF	TNF receptor-associated factor
Treg	regulatory T cell
TUFM	Tu translation elongation factor
UC	ulcerative colitis
UVRAG	UV radiation resistance-associated gene
VSV	vesicular stomatitis virus
XIAP	X-linked inhibitor of apoptosis
